# Dying Cells Protect Survivors from Radiation-Induced Cell Death in *Drosophila*

**DOI:** 10.1371/journal.pgen.1004220

**Published:** 2014-03-27

**Authors:** Amber Bilak, Lyle Uyetake, Tin Tin Su

**Affiliations:** Department of Molecular, Cellular and Developmental Biology, University of Colorado, Boulder, Colorado, United States of America; The University of North Carolina at Chapel Hill, United States of America

## Abstract

We report a phenomenon wherein induction of cell death by a variety of means in wing imaginal discs of *Drosophila* larvae resulted in the activation of an anti-apoptotic microRNA, *bantam*. Cells in the vicinity of dying cells also become harder to kill by ionizing radiation (IR)-induced apoptosis. Both *ban* activation and increased protection from IR required receptor tyrosine kinase Tie, which we identified in a genetic screen for modifiers of *ban*. *tie* mutants were hypersensitive to radiation, and radiation sensitivity of *tie* mutants was rescued by increased *ban* gene dosage. We propose that dying cells activate *ban* in surviving cells through Tie to make the latter cells harder to kill, thereby preserving tissues and ensuring organism survival. The protective effect we report differs from classical radiation bystander effect in which neighbors of irradiated cells become more prone to death. The protective effect also differs from the previously described effect of dying cells that results in proliferation of nearby cells in *Drosophila* larval discs. If conserved in mammals, a phenomenon in which dying cells make the rest harder to kill by IR could have implications for treatments that involve the sequential use of cytotoxic agents and radiation therapy.

## Author Summary

In multicellular organisms where cells exist in the context of other cells, the behavior of one affects the others. The consequences of such interactions include not just cell fate choices but also life and death decisions. In the wing primordia of *Drosophila melanogaster* larvae, dying cells release mitogenic signals that stimulate the neighbors to proliferate. Such an effect is proposed to compensate for cell loss and help regenerate the tissue. We report here that, in the same experimental system, dying cells activate a pro-survival microRNA, *bantam*, in surviving cells. This results in increased protection from the killing effect of ionizing radiation (IR). Activation of *ban* requires *tie*, which encodes a receptor tyrosine kinase. *tie* and *ban* mutant larvae are hypersensitive to killing by IR, suggesting that the responses described here are important for organismal survival following radiation exposure.

## Introduction

In metazoa where cells exist in the context of other cells, the behavior of one affects the others. The consequences of such interactions include not just cell fate choices but also life and death decisions. In wing imaginal discs of *Drosophila melanogaster* larvae, dying cells release mitogenic signals [1], [2], [3]. Signaling from dying cells, or dying cells kept alive by the caspase inhibitor p35 (the so-called ‘undead’ cells), in wing discs operate through activation of Wingless (*Drosophila* Wnt) and JNK, and through repression of the tumor suppressor Salvador/Warts/Hippo pathway. A crosstalk between JNK and Hpo has also been reported [1]. The consequences on the neighbors include increased number of cells in S phase and activation of targets of Yki, a transcription factor that is normally repressed by Hpo signaling [3]. Mitogenic signals from dying cells results in increased proliferation of neighbors, which is proposed to compensate for cell loss and help regenerate the disc.

A target of Yki is *bantam* microRNA [4], but *ban* was not examined in above-described studies. *ban* was first uncovered in a genetic screen for promoters of tissue growth when overexpressed in *Drosophila* [5]. Further study found a role for *ban* in both preventing apoptosis and promoting proliferation [6]. A key target of *ban* in apoptosis is *hid,* a *Drosophila* ortholog of mammalian SMAC/Diablo proteins. These proteins antagonize DIAP1 to liberate active caspases and allow apoptosis. Hid is pro-apoptotic; repression of Hid by *ban* via binding sites in *hid* 3′UTR curbs apoptosis [6].

Since the initial characterization of *ban*, the role of this miRNA has expanded to include coordinating differentiation and proliferation in neural and glial lineages, cell fate decisions in germ line stem cells, in circadian rhythm, and in ecdyson hormone production [7], [8], [9], [10], [11]. In these and other contexts, *ban* is regulated by a number of transcriptional factors and signaling pathways including, Hpo/Yki, Wg, Myc, Mad, Notch and Htx [6], [12], [13], [14], [15]. The regulatory region of *ban* gene is likely to be complex and substantial; p-element insertions more than 10 kb away from *ban* sequences produce *ban* phenotypes [5].

The experimental evidence in *Drosophila* that dying cells promote proliferation presaged by several years the experimental evidence for a similar but mechanistically different phenomenon in mammals. A response called ‘Phoenix Rising’ occurs in mice after cell killing by ionizing radiation. Here, the activity of Caspase 3 and 7 is required in dying cells and mediates the release of prostaglandin E_2_, a stimulator of cell proliferation [16]. These signals act non-autonomously to stimulate proliferation and tissue regeneration. A follow-up study in mice found a requirement for Caspase 3 in tumor regeneration after radiation treatment [17]. Not all consequences on neighboring cells are protective or mitogenic. In the classical ‘radiation bystander effect’, seen in cell culture and in mice, the effect of irradiated cells on the neighbors is destructive, making the latter more prone to death [18], [19], [20]. There is evidence for a soluble signal; media from irradiated cells can induce the bystander effect on naïve cells. Inhibitors of the bystander effect include antioxidants [21], suggesting that oxidative stress and energy metabolism may be involved in radiation bystander effect.

We showed previously that *ban* activity increased after exposure to ionizing radiation (IR) in wing imaginal discs of *Drosophila* larvae [22]. IR-induced increase in *ban* activity required caspase activity: expression of a viral caspase inhibitor, p35, or mutations in p53 that reduced and delayed the onset of caspase activation attenuated *ban* activation. We noted that while IR-induced cell death was scattered throughout the disc, *ban* activation was homogeneous. This suggested a non-cell-autonomous component in activation of *ban*. The current study came out of our efforts to understand how *ban* is activated in response to IR. We identified *Drosophila tie*, which encodes a receptor tyrosine kinase of VGFR/PDGFR family, as an important mediator of IR-induced changes in *ban*. Previous knowledge of Tie function in *Drosophila* was limited to long range signaling for border cell migration during oogenesis [23]. We report here that Tie was needed to activate *ban* in response to cell death. One consequence of *ban* activation, we found, was that remaining cells were harder to kill by IR.

## Results

### *bantam* activation in response to cell killing by IR or clonal cell death

To detect *ban* activity, we used a published GFP transgene that is expressed from the tubulin promoter and includes 2 perfect *ban* target sequences in its 3′UTR, allowing for repression by *ban* [6]. We reproduced our published data that *ban* activity increased in wing discs after exposure to IR [22] (Fig. S1). A published control sensor that lacks sequences for *ban* binding did not show a change in GFP under the same conditions (Fig. S1 ‘control sensor’).

**Figure 1:**
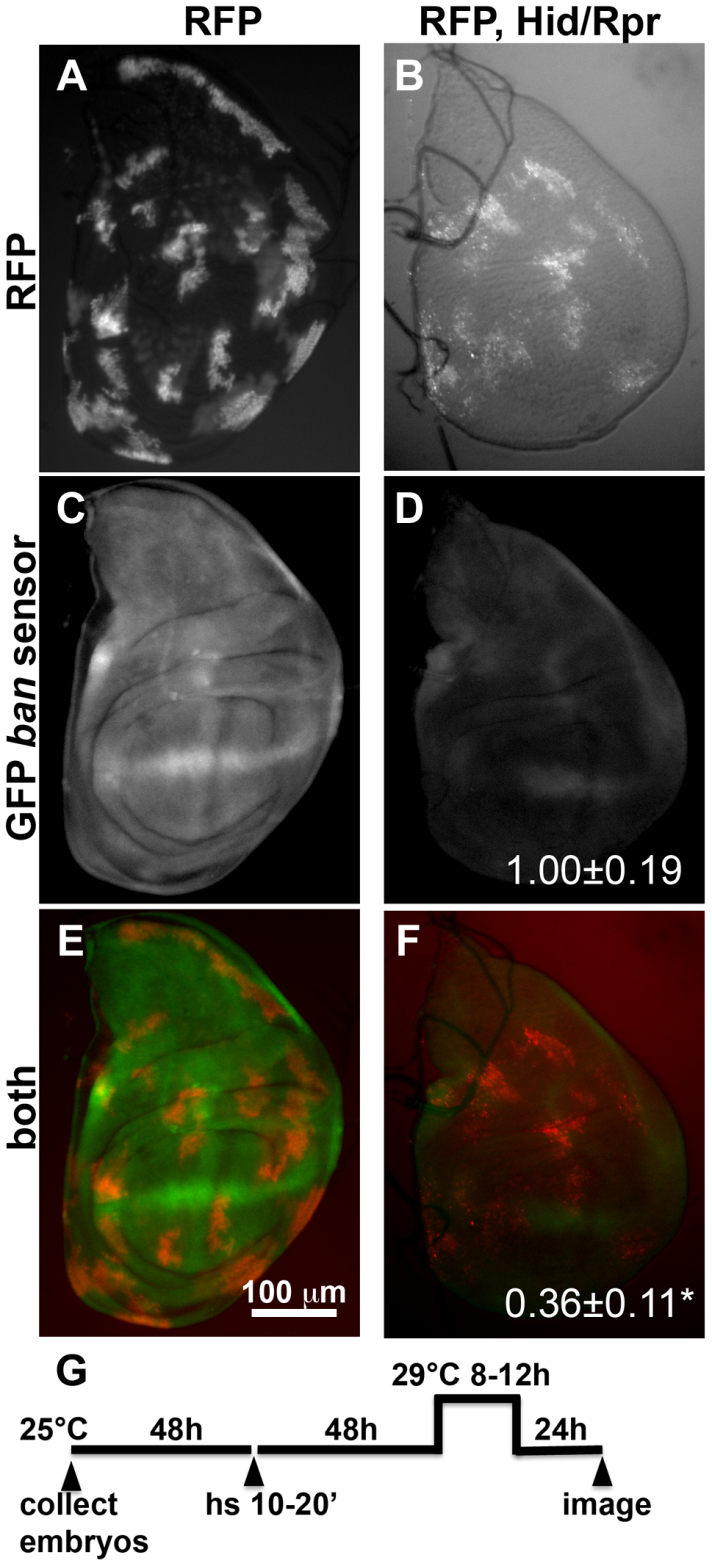
Activation of *ban* after clonal induction of cell death in wing discs. The larvae were heat-shocked (hs) to induce FLP-mediated excision of intervening sequences to allow GAL4 expression from the actin promoter. After heat-shock, *actin-GAL4* was kept repressed with *tubulin-GAL80^ts^* to allow clones to form. A shift to 29°C inactivated GAL80 and de-repressed GAL4, which drove the expression of UAS-*hid,* UAS*-rpr* and the UAS-*RFP* clonal marker. (A, C, E) Wing discs from a control RFP only larva. (B, D, F) Wing discs from an RFP, *hid, rpr* larva. The discs were imaged live for RFP and GFP. (G) shows the timeline followed. GFP images in C and D were acquired and processed using identical parameters to allow comparison. RFP was weaker in Hid/Rpr clones than in control clones; therefore RFP images such as those in B were visualized with increased brightness. The mean GFP signal was quantified for each disc, normalized to the average for controls (RFP only) discs and shown on panels D and F. *p<0.001. ‘RFP’  =  *hs-FLP/Y;* GFP ban sensor*/+;tub-GAL80^ts^/Act>>GAL4,* UAS*-RFP*. ‘RFP, Hid/Rpr’ carries the same transgenes and has UAS-*hid*, UAS-*rpr* instead of Y. ‘h’  =  hour or hours.

Next, we investigated whether the *ban* sensor was responsive to cell killing by another method; we were concerned that although only some cells died in irradiated discs, all were exposed to a death-inducing DNA-damaging agent that might have caused changes in *ban*. FLP-recombinase mediated ‘flip-out’ method was used to express GAL4 in random, scattered cells in 3^rd^ instar larval wing discs. GAL4 then drove the expression of pro-apoptotic genes, *hid* and *reaper,* to result in cell kill. In these experiments, FLP was driven from a heat shock promoter using a brief (10–20′) heat pulse at 37°C. Constitutive expression of GAL80^ts^ from a tubulin promoter kept GAL4 repressed, and allowed clones to form. To induce cell death, GAL80 was inactivated by an incubation at 29°C for 8–12 h (10′ heat shock and 8 h incubation at 29° shown in Fig. 1). Cells expressing GAL4 were marked with RFP. In irradiated discs, a drop in GFP from the sensor was most prominent at >20 h after robust cell death [22]; this was expected because GFP has a half-life of 26 h [24]. Therefore, we monitored the sensor 22–24 h after de-repression of GAL4 (Fig. 1G). We found that GFP was reduced in discs with Hid/Rpr clones compared to discs with RFP-only clones (Fig. 1C vs. D). In discs with flip-out clones, surviving cells had not been exposed to a death-inducing DNA-damaging agent. Yet, these also showed a drop in sensor, reflecting *ban* activation. Co-expression of p35 with Hid/Rpr in clones prevented the decrease in GFP (Figure S1). This is in agreement with our published finding that p35 also prevented a decrease in the GFP *ban* sensor in irradiated discs [22].

### *bantam* activation in response to cell killing in the *ptc* domain

In irradiated wing discs or in wing discs with Hid/Rpr clones, cell death was scattered but GFP *ban* sensor decreased throughout the disc. We infer that at least some instances of *ban* activation occurred in non-apoptotic cells, that is, non-autonomously. To address this idea more rigorously, we killed cells in a known, defined, and invariant location in wing discs.

**Figure 2:**
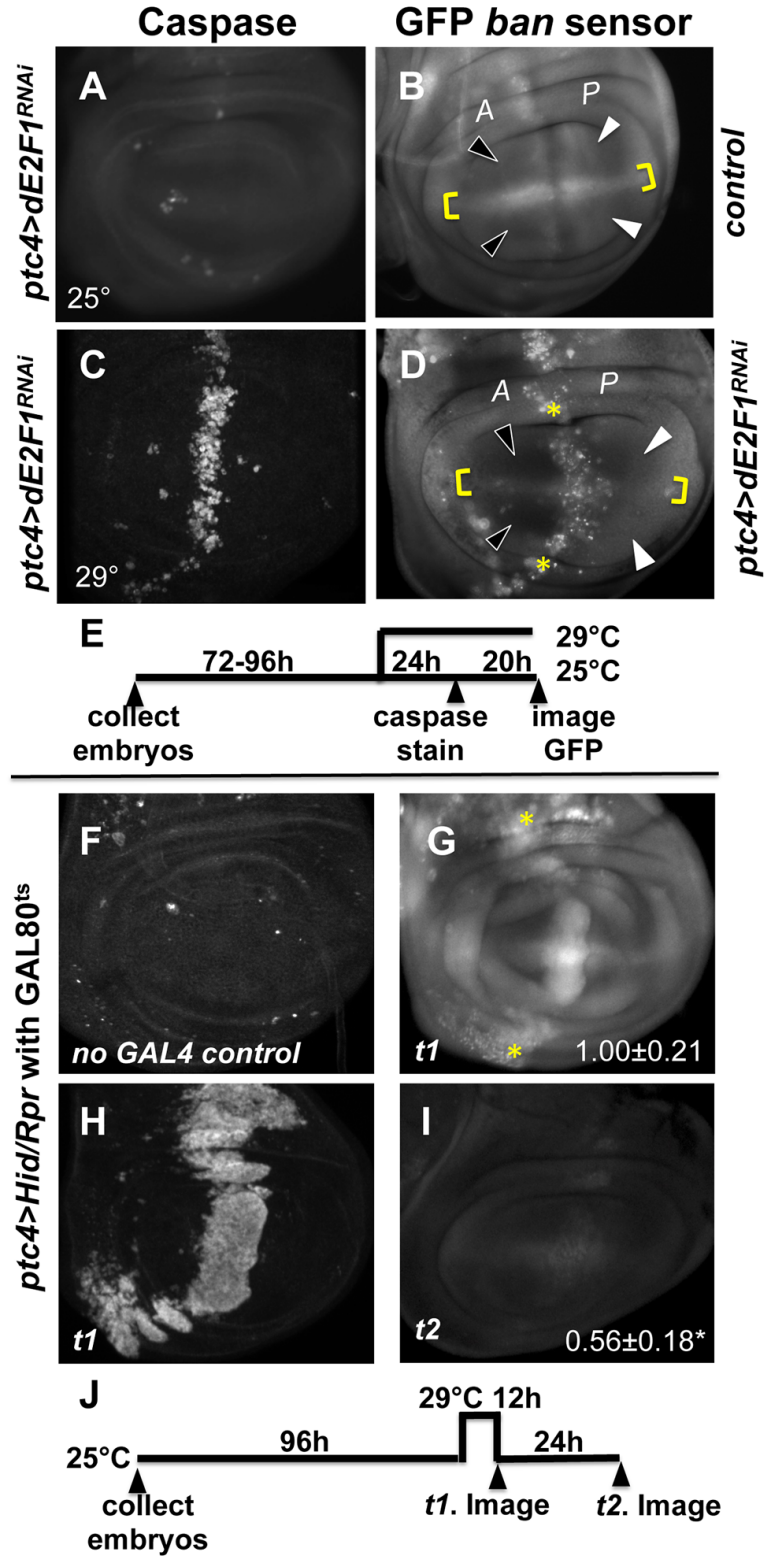
Changes in GFP *ban* sensor in discs with *ptc-GAL4*-driven cell death. (A–D) Larvae carrying one copy each of *ptc-GAL4, UAS-ds RNA against dE2F1 (ptc4>dE2F1^RNAi^*) and the GFP *ban* sensor were raised at 25°C for 72–96 hours and either maintained at the same temperature (A, B) or shifted to 29°C for 24 h (C, D). Wing imaginal discs were fixed and stained for cleaved Caspase 3 (A, C) or imaged live for GFP *ban* sensor (B, D). (E) shows the timeline followed. Embryos were collected for 4–6 hours. *A* =  Anterior; *P*  =  Posterior. Cell death was minimal at 25°C (A) but prominent after the temperature shift (C). Cells anterior to the *ptc* domain (black arrowheads) showed lower GFP compared to cells posterior (white arrowheads) in (D) but not in (B). The ratio of GFP fluorescence in *A* and *P* compartment for each disc was quantified using Image J, and the averages are shown in Table 1. Only the pouch regions were considered. (F–I) Larvae carrying one copy each of *ptc-GAL4, UAS-hid, UAS-rpr, tub-GAL80^ts^* and the GFP *ban* sensor were raised at 25°C for 96 hours and shifted to 29°C to inactivate GAL80. (J) shows the timeline followed. Embryos were collected for 4–6 hours. Wing imaginal discs were fixed and stained for cleaved Caspase 3 (F, H) or imaged live for GFP *ban* sensor (G, I). Cell death was absent in ‘no GAL4’ controls (F) but robust after de-repression of GAL4 (H). *ban* sensor was bright immediately after de-repression of GAL4 for 12 hours (G, *t1* time point), but was reduced throughout such discs 24 hours later (I, *t2* time point). The mean GFP signal was quantified for each disc, normalized to the average for discs at *t1* and shown on panels G and I. Dying cells in these discs sometimes, but not always, displayed elevated GFP (* in D and G); we do not know the reason.

Expression of a dsRNA against *de2f1* under the control of *patched-GAL4* (to be called ‘*ptc4>dE2f1^RNAi^*’) reduced E2F1 protein levels and the expression of an E2F1 target reporter in a stripe of anterior compartment cells along the Anterior/Posterior (A/P) compartment boundary of wing discs [25]. And the corresponding region in the adult wings was reduced in size. Mutations in dE2F1 caused cell-autonomous apoptosis [26]. Therefore, we asked whether depletion of dE2F1 by RNAi also killed cells. We detected little apoptosis in wing discs of *ptc4>dE2f1^RNAi^* 3^rd^ instar larvae raised at 25°C (Fig. 2), even though we could see the expression of a UAS-eGFP reporter in the same larvae (Fig. S2). Presumably, RNAi-mediated knockdown of dE2F1 was sub-optimal under these conditions. A temperature-dependence in the effect of *ptc4>dE2f1^RNAi^* on the size of the inter-vein region of the adult wing was noted before [25]. Therefore, in an attempt to enhance the killing effect of dE2f1^RNAi^, we shifted the larvae from 25°C to 29°C, at 72 h after egg deposition (AED). We observed robust caspase activity in the *ptc* domain 24 hours (h) after the temperature shift (Fig. 2C). Active caspase staining was stronger in the pouch than the rest of the disc; therefore, we focused on the pouch in subsequent analyses of the consequence of cell death. Similar results were seen by shifting larvae at 96 h AED instead of 72 h. At these times, *ptc* domain had already narrowed to a vertical stripe [Fig. S2 in [27]; Fig. S2].

To see if *ptc4>dE2f1^RNAi^*-induced cell death resulted in changes in *ban* activity, we monitored GFP *ban* sensor after an additional 20 h to allow for the half-life of GFP, that is, at 44 h after temperature shift. In wing discs from control larvae without *ptc4>dE2f1^RNAi^*, pouch cells in the Anterior (A) and the Posterior (P) compartments showed similar GFP signals, producing an A/P ratio of 1 (Fig. 2B, quantified in Table 1). A stripe of cells at the dorsal/ventral (D/V) boundary showed high sensor signal (low *ban* activity; between brackets). This was noted before and represents repression of *ban* by *N/wg* along the D/V boundary [6], [15]. In wing discs from *ptc4>dE2f1^RNAi^* larvae, cells anterior to the *ptc* domain showed lower GFP signal than cells posterior to the *ptc* domain (Fig. 2D, black vs. white arrowheads; quantified in Table 1). This reduction was small, reducing the A/P ratio by about 20%, but was reproducible and bore physiological consequences as described later. The P compartment of some but not all *ptc4>dE2f1^RNAi^* discs also showed signs of reduced GFP (Fig. 2D and B, white arrows). But this difference was weaker than the change in the A compartment (thus producing A/P ratios <1 in discs with *ptc4>dE2f1^RNAi^*) and not reproducible. Shifting larvae to 29°C at 96 h AED instead of 72 h produced similar results. We conclude that cell death in the *ptc* domain resulted in a localized decrease in GFP *ban* sensor, reflecting activation of *ban*.

**Table 1:** Mean fluorescence in the wing pouch (Anterior normalized to Posterior).

assay	genotype	A/P	STD	p value	p value relative to	n
GFP	ban sensor/+	1.00	±0.08	<0.001	PE3/ban sensor	8
GFP	PE3/ban sensor	0.80	±0.09	N/A	N/A	12
GFP	PE3/control sensor	1.03	±0.10	<0.001	PE3/ban sensor	8
GFP	PE3/+; tie{Df}/+	0.90	±0.09	<0.05	PE3/ban sensor	11
caspase	+/+ (yw)	1.03	±0.14	<0.001	PE3/+	8
caspase	PE3/+	0.60	±0.11	N/A	N/A	25
caspase	PE3/+; ban{D1}/+	0.86	±0.18	<0.001	PE3/+	6
caspase	PE3/banA; ban{ D1}/+	0.60	±0.15	0.002	PE3/+; ban{ D1}/+	15
caspase	PE3/+; tie{Df}/+	0.87	±0.18	<0.001	PE3/+	10
caspase	PE3/banA; tie{Df}/+	0.67	±0.08	<0.01	PE3/+; tie{Df}/+	9
caspase	Pvf1{EP1624}; PE3/+	0.86	±0.12	<0.001	PE3/+	14
TUNEL	+/+ (yw)	0.95	±0.22	<0.001	PE3/+	7
TUNEL	PE3/+	0.47	±0.16	N/A	N/A	11

N  =  number of discs examined. ‘PE3’  =  *ptc-GAL4>UAS-dsRNA* against dE2F1.

Expression of UAS-Hid/Rpr from the *ptc-GAL4* driver also produced cell death. In fact, we needed to keep GAL4 repressed with GAL80^ts^ in order to allow survival to 3^rd^ instar. De-repression of GAL4 by incubation at 29°C for 12 h was sufficient to induce robust cell death in the *ptc* domain (Fig. 2H). This was followed by a decrease in GFP throughout the disc 24 h later (Fig. 2I). We conclude that with more robust cell death in the *ptc* domain, a greater area of the disc activated *ban*.

In some wing discs expressing *ptc4>dE2f1^RNAi^,* bright specks of GFP were seen in the *ptc* domain as cells underwent apoptosis (e.g. * in Fig. 2D). Elevated GFP was also seen in the dying cells in *ptc4>Hid/Rpr* discs immediately after GAL4 de-repression (* in Fig. 2G) but not at later times (Fig. 2I), and rarely in irradiated discs with cell death (Fig. S1). We do not know the reason for elevated GFP in dying cells in some of these cases, but we emphasize that a decrease in the sensor occurred in the survivors outside the domain of cell death in all cases, which we focused on in subsequent sections.

### Apoptosis in the *ptc* domain accompanied resistance to IR in the surviving cells

*ban* is anti-apoptotic and acts by repressing Hid expression [6]. *hid,* along with *rpr* and *skl* are induced by radiation in a p53-dependent manner (e.g. [28], [29]). Hid, Rpr and Skl antagonize DIAP1 to activate caspases and induce apoptosis. Reduction of *hid* gene dosage by half has been shown to reduce IR-induced apoptosis [28], [29].

**Figure 3:**
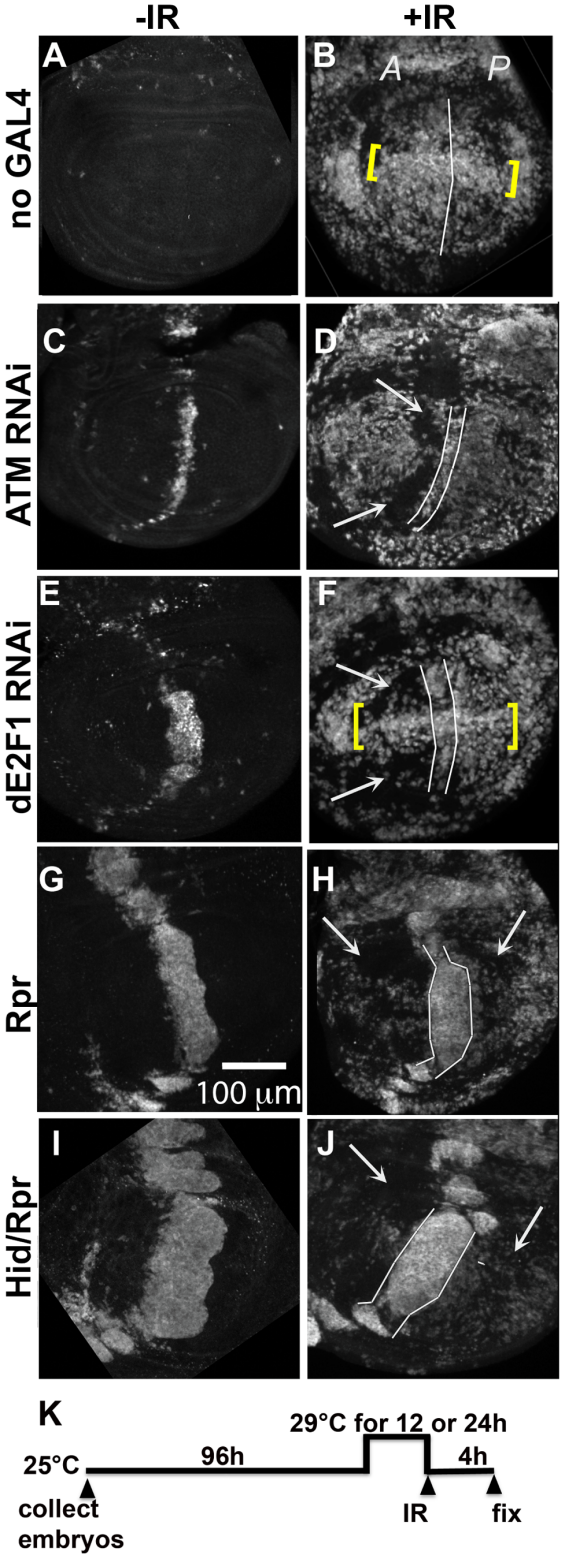
Cells in discs with prior cell death are resistant to IR-induced cell death. Wing imaginal discs were extirpated from third instar larvae 4(-IR) or 4000 R (+IR) of X-rays, fixed and stained for cleaved Caspase 3. Only the pouch regions are shown. *A* =  Anterior. *P* =  Posterior. (A–B) control discs without GAL4. (B) IR-induced caspase staining was similar in the *A* and *P* halves of the pouch in *y^1^w^1118^* discs. Cells along the D/V boundary where *ban* sensor is high, reflecting low *ban* activity, were more sensitive to IR-induced death (yellow brackets). (C–F) discs from larvae with one copy of *ptc-GAL4* and one copy of UAS-ds RNA against ATM (C,D) or dE2F1 (E, F). Areas with reduced IR-induced caspase activity (arrows) were found anterior to the domain of cell death induced by *ptc-GAL4* (between lines). Cells along the D/V boundary were refractory to the protection (yellow brackets in F). (G–J) discs from larvae with one copy of *ptc-GAL4* and one copy of UAS-*rpr* (G, H) or UAS-*hid*, UAS-*rpr* (I, J). These larvae also carried one copy of *tub-GAL80^ts^*. The protected areas (arrows) included most of the A compartment and parts of the P compartment in (H). The protected area was even greater in (J). We note that death in the *ptc* domain in Hid/Rpr discs was wider in –IR discs than in +IR discs. These cells seemed to protrude from the disc after irradiation, which could explain the narrowing of the domain. (K) shows the timeline followed. Embryos were collected for 4–6 hours. The duration of incubation at 29°C was 24 hours for ATM or dE2F1 RNAi larvae and 12 hours for Rpr or Hid/Rpr larvae.

If *ban* was activated in the surviving cells in the experiments described above, the former may show altered sensitivity to apoptotic-inducing stimuli. To test this possibility, we irradiated larvae with *ptc-GAL4*-driven cell death and monitored IR-induced cell death using an antibody against cleaved Caspase 3. In *y^1^w^1118^* control discs (Fig. 3B), there was robust cell death in both A and P compartments of the wing pouch. Along the D/V boundary, low *ban* activity correlated with high caspase activity as expected (brackets).

To test for any protective effects of cell death, we used two additional death-inducing constructs. Loss of *Drosophila* ATM, encoded by *telomere fusion*, resulted in cell autonomous apoptosis due to telomere dysfunction and activation of the DNA damage responses [30]. This method removed complications due to reduced dE2F1. Cell killing by ATM^RNAi^, we found, was not as robust as cell killing by dE2f1^RNAi^ (Fig. 3C vs. E; Fig. S2). We also used *UAS-Rpr* to induce death by *ptc-GAL4*. As in the case of *UAS-Hid/Rpr*, *UAS-Rpr* also had to be kept repressed with GAL80^ts^ to allow larval survival to 3^rd^ instar and was able to induce cell death with a 12 h de-repression of GAL4. But the resulting cell death was less robust than *UAS-Hid/Rpr* under the same experimental conditions (Fig. 3 G vs. I).

Irradiation of these discs showed that prior cell death in the *ptc* domain accompanied a reduction in IR-induced caspase activation (Fig. 3D, F, H and J arrows). The protected area was localized in D and F but expanded in H and J, showing an increase with increasing level of prior death. In discs expressing dE2f1^RNAi^ (Fig. 3F) or Hid/Rpr (Fig. 3J), the protected area corresponded to the area with reduced *ban* sensor (Fig. 2D and I). We conclude that prior cell death in the *ptc* domain resulted in *ban* activation and protection from IR-induced cell death in the rest of the disc, in a dose-dependent manner. Cells in the same compartment as the dying cells (A compartment) were the first to benefit but the protective effect was able to reach the P compartment with increased *ptc-GAL4*-driven cell death in the case of Rpr and Hid/Rpr (compare Fig 3J to B).

**Figure 4:**
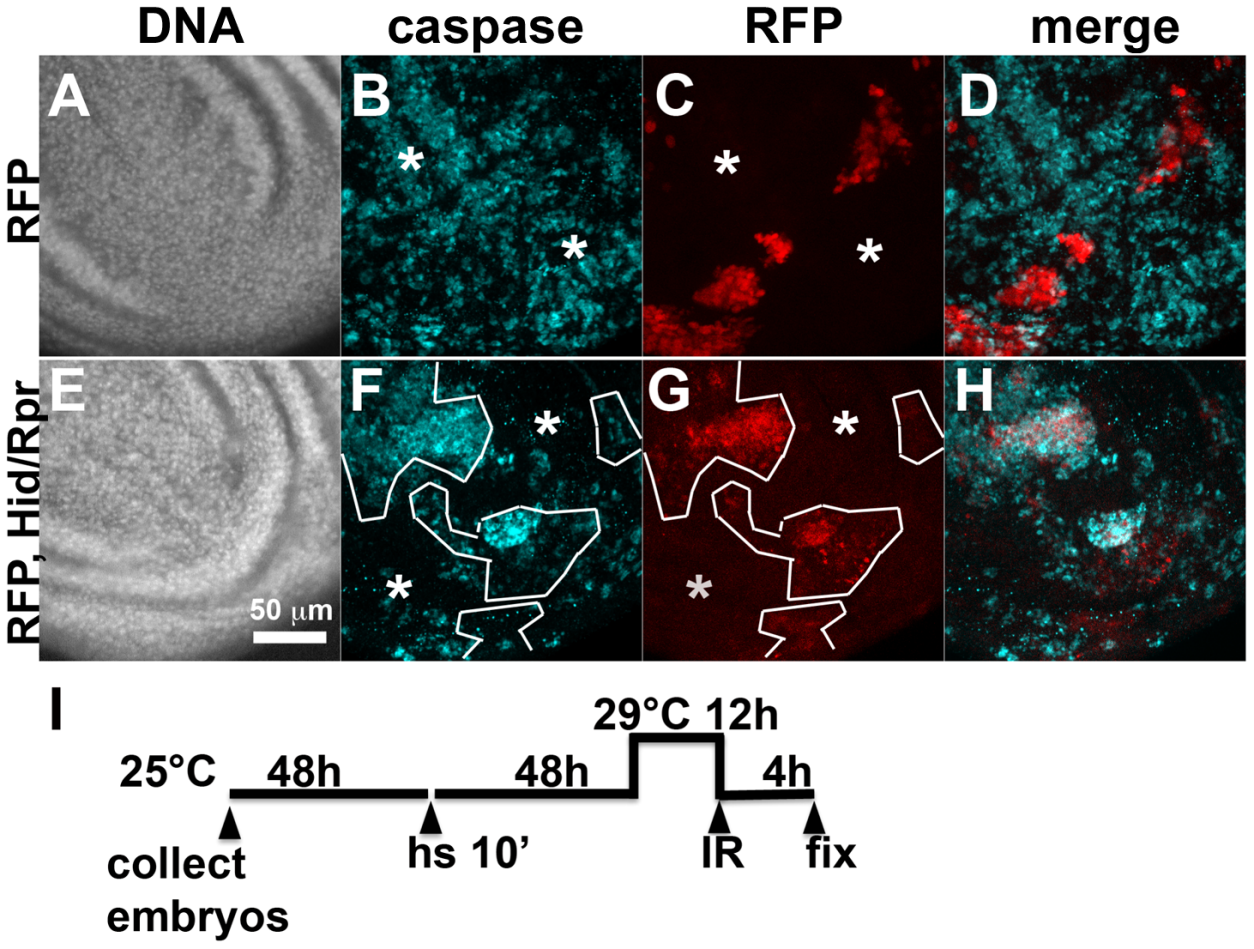
Cell death and protection in discs with clonal Hid/Rpr expression. The larvae were generated as described for Figure 1 and irradiated with 4000R of X-rays after de-repression of GAL4. Wing imaginal discs were extirpated 4 h after irradiation and fixed and stained for DNA (A, E) and cleaved active Caspase 3 (B, F). RFP clonal marker (C, G) was used to mark the clonal boundaries in the images. (D, H) show merged images. (I) shows the timeline followed. * shows areas outside the clone that display robust caspase activation in RFP only discs (top row) but not in RFP, Hid/Rpr discs (bottom row).

**Figure 5:**
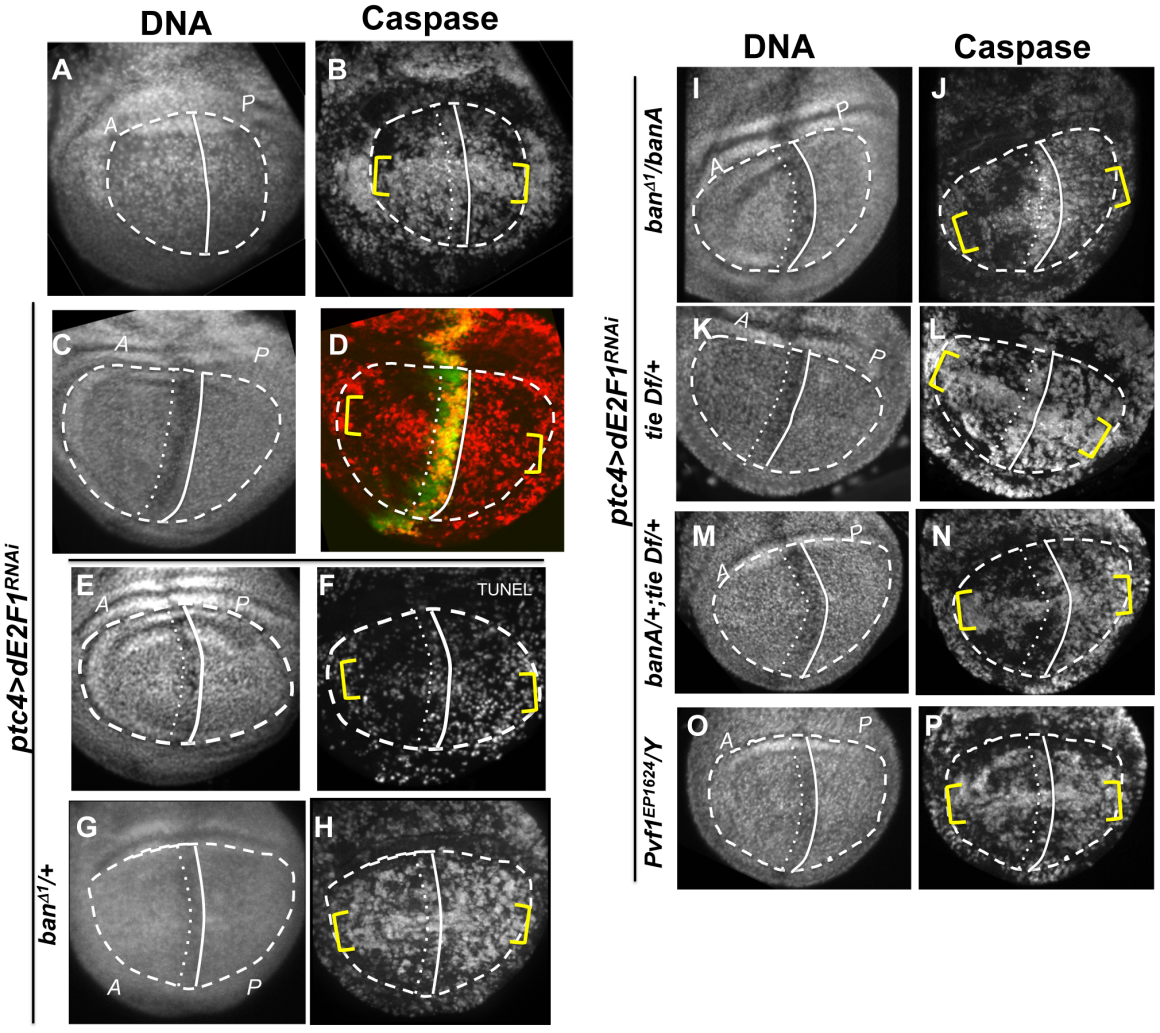
The effect of *ptc>dE2F1^RNAi^* is modified in different genetic backgrounds. Wing imaginal discs from larvae carrying one copy each of *ptc4-GAL4* and UAS-dsRNA against dE2F1 in different mutant backgrounds were fixed and stained for cleaved Caspase 3 (as indicated) or processed for TUNEL (F) at 4 h after irradiation with 4000R of X-rays. The discs were also stained for DNA. DNA stained images were used to locate the pouch (within the dashed line), the A/P boundary (solid vertical line) and the *ptc* domain (between vertical lines). *UAS-GFP* (green) in (D) confirmed the location of the *ptc* domain. Yellow brackets indicate apoptosis along the presumed D/V boundary in some panels. The experimental timeline in Fig. 3 K was followed, using a 24 h incubation at 29°C to de-repress GAL4. Embryos were collected for 4–6 hours. The caspase or TUNEL signal from A and P compartments of the pouch, exclusive of the *ptc* domain, were quantified from images such as these and shown in Table 1. (A–B) *y^1^w^1118^* discs reproduced from Fig. 3B, for comparison. (C-P) All larvae carried one copy each of *ptc-GAL4* and UAS-dsRNA against dE2F1. The larva in (C,D) also carried one copy each of *tub-GAL80^ts^* and *UAS-GFP*. Additional genotypes are indicated next to the panels. banA =  UAS-banA.

To rule out the possibility that the protection is due simply to disruptions in the *ptc* domain, we induced cell death in random clones; this too resulted in protection (Fig. 4, described in detail below). To rule out unwanted consequences of depleting dE2F1 earlier in development, we repeated the experiments with *ptc4>dE2f1^RNAi^* but used GAL80t^ts^ to repress GAL4 until 96 h AED. GAL4 was then de-repressed for 24 h and protection from IR-induced apoptosis assayed (Fig. 5D). The results were indistinguishable from what we saw without GAL80 (Fig. 3F). We also ruled out the possibility that dE2f1^RNAi^ in the *ptc* domain altered cell proliferation in the pouch to interfere with IR-induced cell death; we saw no change in the mitotic index in A and P compartments in discs with *ptc4>dE2f1^RNAi^* (Fig. S3). We also ruled out the possibility that death in the *ptc* domain interfered with developmental signaling; we saw no change in Dpp-lac Z reporter expression in control and *ptc4>dE2f1^RNAi^* discs (Fig. S4).

### Death in Hid/Rpr flip-out clones also resulted in protection from IR

Death within the *ptc* domain accompanied increased *ban* activity and protection from IR-induced cell death outside the *ptc* domain. Death in Hid/Rpr clones also increased *ban* activity outside the clones (Fig. 1). Therefore, we asked if Hid/Rpr clones also resulted in protection from IR-induced cell death. Discs like those in Fig. 1C and D were irradiated and IR-induced caspase activity was monitored. We found robust caspase activation in areas outside RFP-only clones (Fig. 4A–D). In contrast, areas outside RFP-marked Hid/Rpr clones showed reduced caspase activation (Fig. 4E–H). We conclude that Hid/Rpr clones also resulted in protection from IR-induced death and that the protected area included cells outside the clones.

### The protective effect of *ptc-GAL4*-induced death is sensitive to *ban* gene dosage

Wing discs expressing *ptc4>dE2f1^RNAi^* showed an intermediate level of protection in the A compartment (Fig. 3F and 5D). The P compartments in these discs showed similar level of caspase activation as the P compartments of controls (Fig. 5B vs. D); the average mean caspase fluorescence in the P compartments was 182±32 arbitrary units for *ptc4>dE2f1^RNAi^* and 163±16 for *yw* in two different experiments. In contrast, the A compartment of *ptc4>dE2f1^RNAi^* pouches show reduced caspase stain compared to P cells in the same disc (Fig. 5D). This allowed us to use the P compartment as an internal control to account for variations in antibody staining, and quantify the reduction in the A compartment in terms of reduced A/P ratio. The *ptc* domain was visible in the DNA images as a ‘depression’ as dead cells were extruded from the cell layer (Fig. 5C). Therefore, we used the DNA images as a guide to locate A and P compartments. The normalized A/P ratio for caspase fluorescence in *ptc4>dE2f1^RNAi^* discs was 0.60±0.11 and was significantly different from the controls (Table 1). TUNEL assay produced similar results (Fig. 5F and Table 1).

This quantitative assay for protection allowed us to test the role of other factors. Co-expression of caspase inhibitor p35 in dying cells attenuated the protective effect (Table 1). This is in agreement with our data that p35 abolished changes in *ban* sensor after irradiation [22] and in discs with Hid/Rpr clones (Fig. S2). Reduction of *ban* gene dosage using heterozygotes of a null allele, *ban^D1^*, attenuated the protection (Fig. 5H and Table 1); this was consistent with the observation that *ban* was activated in the protected regions (Fig. 2D). One copy of the UAS-banA transgene, which encodes the primary transcript and rescued the lethality and growth defects of *ban* null mutants without a GAL4 driver [6], also rescued the protection in *ban^D1^*/+ (Fig. 5J and Table 1).

**Figure 6:**
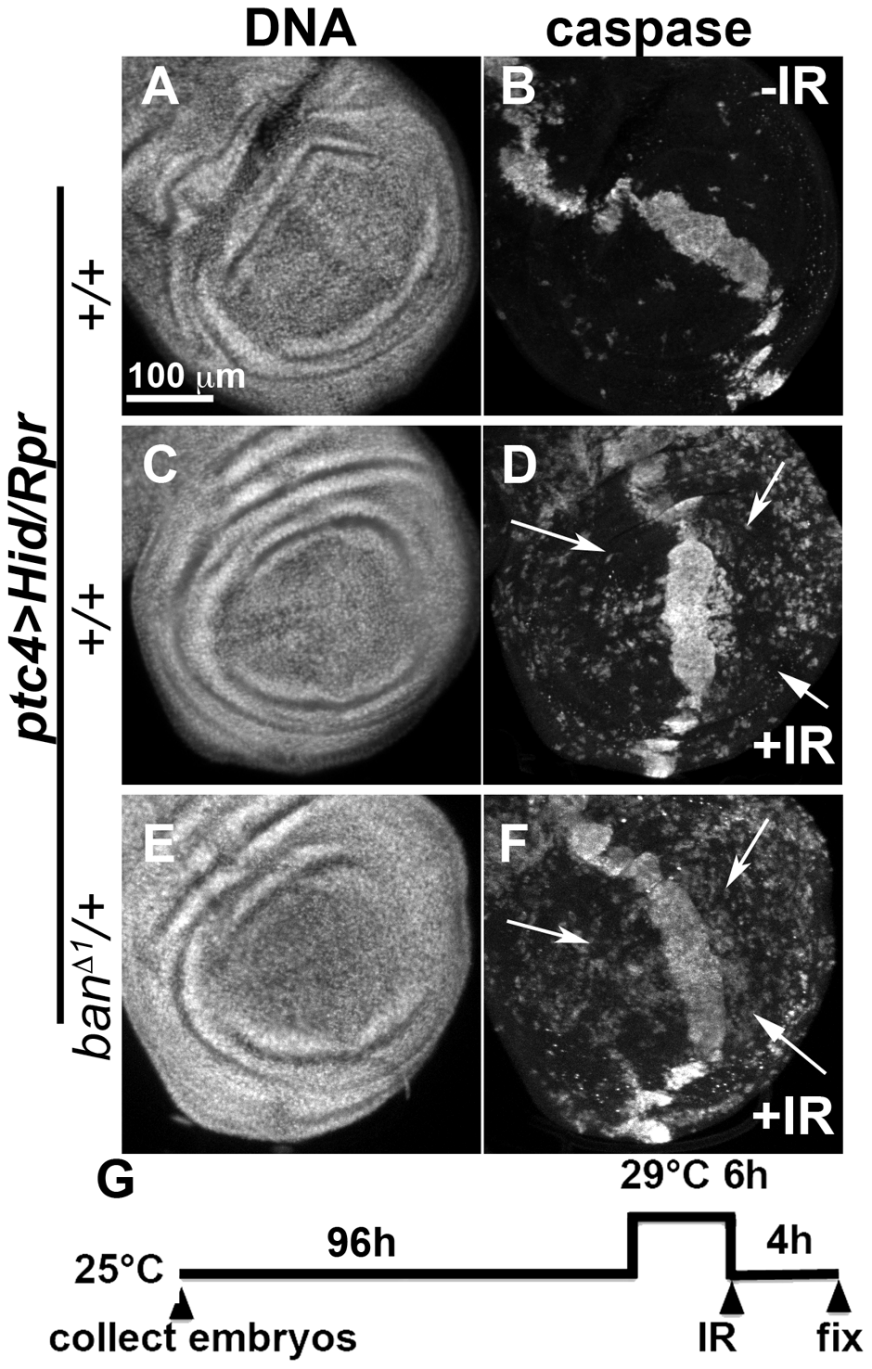
Protection by Hid/Rpr-induced cell death is sensitive to *ban* gene dosage. Wing imaginal discs from larvae carrying one copy each of *ptc4-GAL4*, *UAS-hid, UAS-rpr* and *tub-GAL80^ts^* were fixed and stained for DNA (A, C, E) and cleaved Caspase 3 (B, D, F) at 4 h after irradiation with 0 (-IR) or 4000R (+IR) of X-rays. Additional genotypes were as indicated. (G) shows the timeline followed. In otherwise wild type background (D), areas outside the *ptc* domain showed reduced caspase staining reflecting protection in response to *ptc4>Hid/Rpr* (arrows). In contrast, the corresponding areas in *ban*/+ discs showed caspase activity (F, arrows).

We also addressed the role of *ban* in cell death-induced protection in *ptc4>Hid/Rpr* discs. In initial experiments, we were unable to see significant effects of *ban^D1^*/+ using a protocol in which GAL4 was de-repressed for 12 h (Fig. 3K). We reasoned this could be because the protective effect of Hid/Rpr was too strong to overcome by removing just one copy of *ban*. Removing both copies of *ban* was not an option because we found before that homozygous *ban* mutant cells in the wing disc underwent spontaneous apoptosis [22]. To get around this problem, we de-repressed *ptc4>Hid/Rpr* for 6 h instead of 12 h (Fig. 6). This reduced cell death in the *ptc* domain to the level seen after de-repressing *ptc4*>Rpr for 12 h (compare Fig.6B to Fig. 3G). The protective effect was also reduced (Fig. 6D) and resembled the protection with Rpr de-repressed for 12 h (Fig. 3H) than Hid/Rpr de-repressed for 12 h (Fig. 3J). Importantly, *ban^D1^* heterozygosity further compromised the protection such that we could see caspase activation throughout A and P compartments (Fig. 6F arrows, compare to arrows in 6D). The protection was not completely abolished, however. This could be because protective mechanisms could still act on the remaining copy of *ban*. We conclude that protection by *ptc4>Hid/Rpr* was also sensitive to *ban* gene dosage.

### A screen for modifiers of *ban* identifies *tie*

To better understand the role of *ban* in radiation responses, we performed a forward genetic screen for modifiers of a *ban* phenotype. *ban* mutant larvae are hypersensitive to IR but this sensitivity can be rescued by reducing the gene dosage of *hid*, a known target of *ban* [22]. Using a similar experimental protocol, we sought to identify dominant modifiers of IR sensitivity of *ban* mutant larvae. We used 4000R of X-rays because this dose resulted in an intermediate level of lethality in *ban* mutants such that both suppressors and enhancers may be uncovered.

Chromosomal deficiencies (Df) used in the screen, on their own, could show IR sensitivity and modify *ban* through simple additive effects. To exclude these, we measured the % survival of *ban* mutants and each Df, and used these to compute the expected survival for an additive interaction (Fig. S5A). We then identified *ban*/Df combinations that produced observed survival that was significantly different from the expected, by X^2^ analysis. We focused on deficiencies that showed consistent and statistically significant effect on both *ban^1170^* and *ban^EP3622^* alleles. Of 78 Df that cover 85% of chromosome 3L and part of 3R, 3 were significant modifiers. *Df(3L)9028* and *Df(3L)6115* enhanced and *Df(3L)6086 * suppressed the IR sensitivity of *ban* mutants. We focused our efforts on *Df(3L)9028* because it deleted a single gene, *tie* (Fig. S5B). *tie* encodes a *Drosophila* homolog of mammalian Tie (Tyrosine kinase with Ig and epidermal growth factor homology domain) [31]. We confirmed the deletion by PCR of genomic DNA (data not shown), and also by q-RT-PCR for *tie* expression (Fig. S5C). *Df(3L)9028* is homozygous viable.

**Figure 7:**
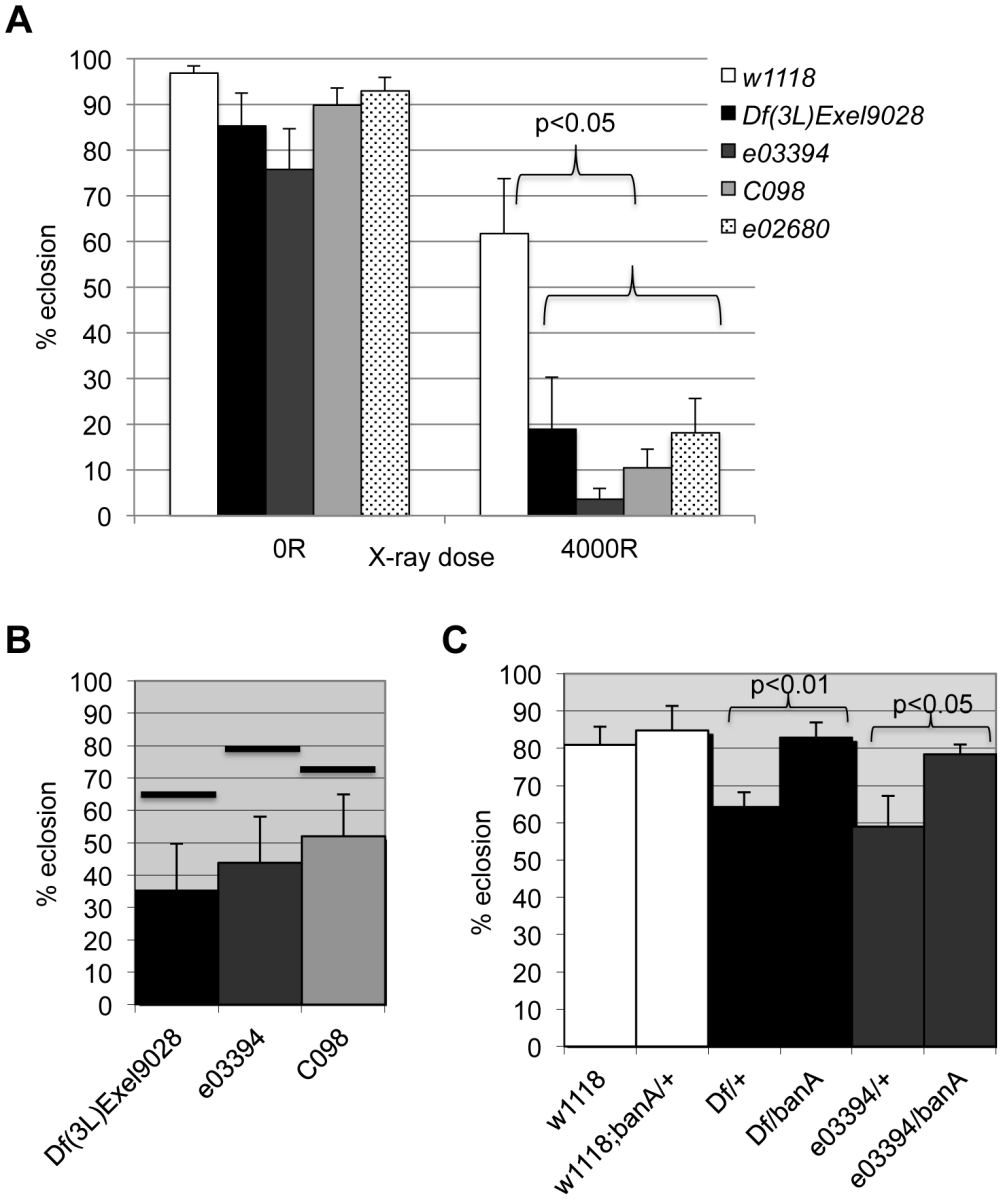
*tie* interacts with *ban* to promote radiation survival. Larvae were irradiated at 96±2 h after egg deposition with 4000R of X-rays. Percent eclosion was determined by counting full and empty pupal cases 10 days after irradiation. Error bar  =  1 SEM. (A) Homozygous *tie* mutants were more radiation sensitive than *w^1118^* controls (p<0.05). N = 106 to 494 pupae per genotype in at least three independent experiments. *tie* alleles were *Df(3L)Exel9028* and transponson insertion alleles in Fig. S5: *tie^e03394^ or ‘03394’, tie^c098^ or ‘co98’ and tie^e02680^ or ‘e02680’.* (B) *tie* and *ban* showed genetic interaction in radiation survival. The observed eclosion of double heterozygotes (*tie/ban^1170^*) is shown for each allele of *tie.* The % eclosion expected from an additive effect between *ban^1170^/+* and *tie/+* was computed from observed % eclosion for each allele and shown as solid horizontal bars above each *tie* allele. In all cases, the observed eclosion was lower than the expected. The difference between expected and observed were significantly different for the three *tie* alleles shown (X^2^ test, p<0.05). N = 94–536 pupae in at least three independent experiments per genotype. (C) A *UAS*-*ban* transgene ‘banA’ rescued the radiation sensitivity of *Df(3L)Exel9028 (‘Df’)* and *tie^e03394^* heterozygotes. N = 359–634 pupae in at least six experiments per genotype.

The data from the screen suggested that *tie* mutants were IR sensitive. We confirmed this using three additional alleles; larvae homozygous of all four alleles show significant and reproducible sensitivity to X-rays (Fig. 7A). Importantly, all three alleles show genetic interaction with *ban* seen with *Df(3L)9028*; each allele, in heterozygous state, reduced the radiation survival of *ban^1170^* heterozygous larvae to levels below what was expected for an additive effect between two mutants (Fig. 7B, data not shown). If *tie* regulates *ban* to promote survival after irradiation, increasing *ban* gene dosage may rescue radiation survival in *tie* mutants. We found that *UAS-banA* did rescue the survival in irradiated *tie* heterozygous larvae (Fig. 7C), but did not change the survival of irradiated *w^1118^* controls in the same experiment (p = 0.6).

### *tie* is needed for IR-induced changes in *ban*

**Figure 8:**
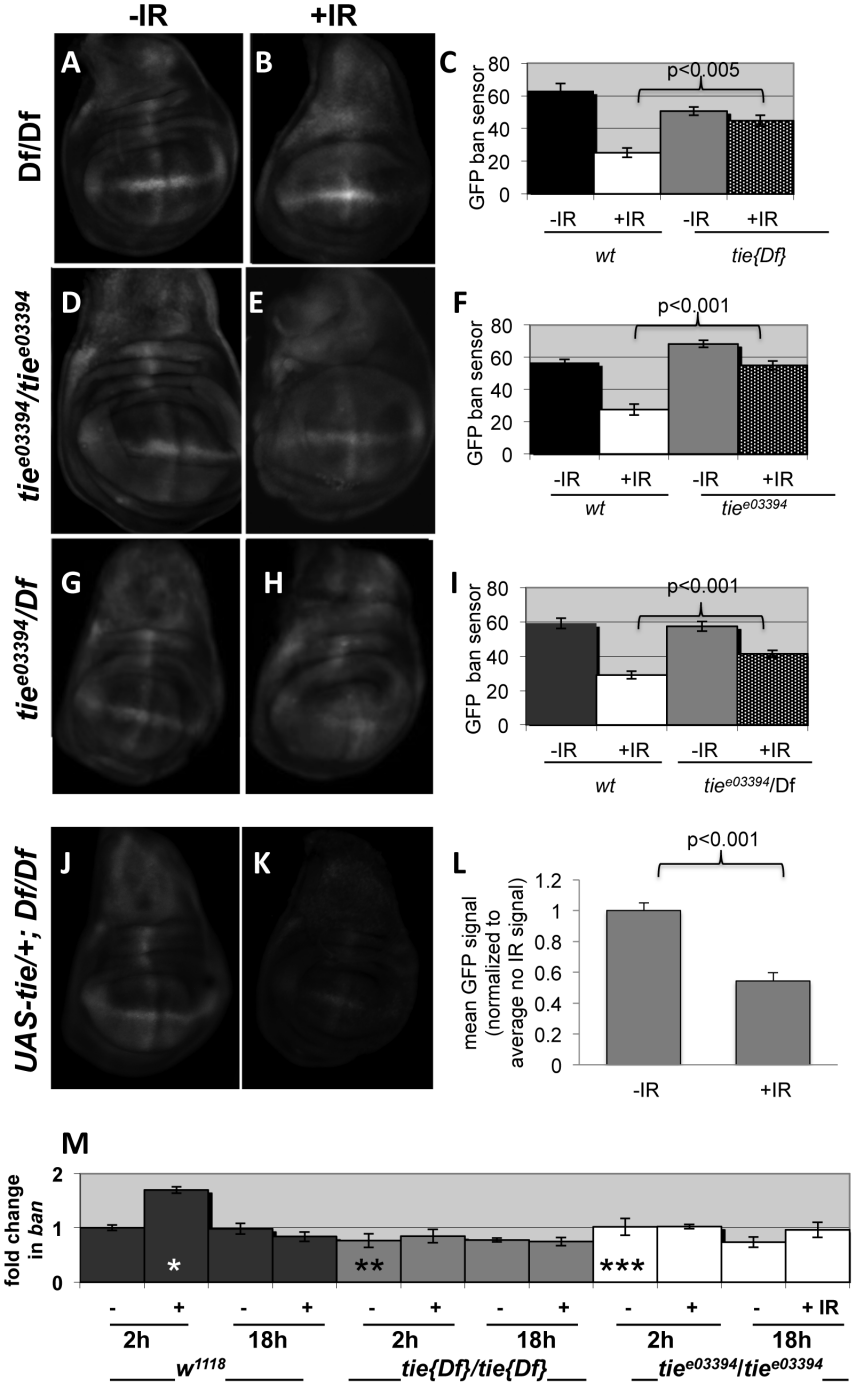
*tie* is needed for IR-induced changes in GFP *ban* sensor. (A–I) Third instar larvae were irradiated at 96±2 hr after egg deposition with 0R (-IR) or 4000R (+IR) of X-rays and wing discs imaged live 24 h later. Images were acquired and treated identically. The mean GFP signal was quantified for each disc using Image J, and the averages are shown for each genotype/treatment. Age-matched sensor only controls (i.e. wild type for *tie*) were included in each experiment (images not shown here but see Fig. S1). (A–C) Wing discs from homozygotes for Df(3L)Exel2098 that removes only *tie* (‘*tie* [*17*]’). N = 31, 28, 31 and 31 discs (left to right) in three independent experiments. (D–F) Wing imaginal discs from homozygotes for *tie^e03394^*. N = 26, 27, 26 and 25 discs (left to right) in three independent experiments. (G–I) Wing imaginal discs from trans-heterozygotes *tie^e03394^/*Df(3L)Exel2098. N = 29, 29, 30 and 30 discs (left to right) in three independent experiments. (J–L) Wing imaginal discs from Df(3L)Exel2098 homozygotes that carry one copy of a *UAS-tie* transgene. The data are from 5 discs per sample in two independent experiments. The larvae carried two copies of the GFP sensor in A-I and one copy in J-L. Error bar =  ±1SEM. p-values reflect a comparison of two samples at the ends of each bracket. (M) *tie* is needed for IR-induced changes in *ban* levels. Quantitative RT-PCR was used to quantify mature *ban* miRNA. The values were normalized to an internal a-tubulin control, and expressed as fold change from un-irradiated *w^1118^* controls (‘w2-’ set at 1). ‘-‘  =  no IR; ‘+’  =  4000R. ‘w’  =  *w^1118^*; Df’  =  homozygotes of Df(3L)Exel2098 that removes only *tie*; ‘3394’  =  *tie^e03394^* homozygotes. *  =  difference compared to w2-. ** and *** =  significance compared to w2+. *  =  p <0.01; **  =  p <0.001; ***  =  p < 10^−6^. Student's t-test was used to determine significance.

The data described so far are consistent with *tie* acting upstream of or parallel to *ban* to confer survival after irradiation. To distinguish between these possibilities, we investigated whether *tie* was needed for IR-induced changes in *ban*. We found that while GFP *ban* sensor decreased after irradiation in control larvae, reflecting activation of *ban* (‘wt’ in Fig. 8C, F and I), this decrease was attenuated in *tie* mutants. All three allelic combination of *tie* studied showed this defect, with Df(3L)9028 homozygotes showing the greatest defect, i.e. the smallest change in GFP between –IR and +IR discs (Fig. 8C). In q-RT-PCR analysis, *tie* was the only gene whose expression was reduced in mutants used here; neither of the flanking genes showed a consistent change in expression (Fig. S5C). In addition, a *UAS-tie* transgene rescued the change in *ban* sensor in Df(3L)9028 homozygotes after irradiation (Fig. 8J-L). Similar to *UAS-banA* [6], the rescue occurred without GAL4 (see also Discussion). Collectively, these data support the idea that *tie* is needed to activate *ban* after irradiation.

Previous work showed that IR-induced caspase activity was needed for *ban* activation following irradiation [22]. Therefore, it is possible that *tie* mutants could not activate *ban* because they could not activate caspases. We do not favor this possibility, however, because we found that wing discs from *tie* mutant larvae were capable of activating caspases after irradiation, as were *tie* homozygous mutant clones of cells (Fig. S6). We conclude that *tie* is not required for IR-induced apoptosis. Because both *tie* and caspase activity were required to activate *ban* but *tie* was not required to activate caspase activation, we conclude that *tie* acts after caspase activation to activate *ban*.

To address how *ban* was activated, we monitored mature *ban* miRNA levels by q-RT-PCR. *ban* increased 2-fold at 2 h after irradiation and returned to control levels by 18 h (Fig. 8M). This timing was consistent with the change in GFP *ban* sensor at 20+ hours after irradiation, given that the half-life of GFP is 26 h. The IR-induced increase in *ban* was abolished in homozygotes of two *tie* alleles examined, suggesting that *tie* was required to increase *ban* levels after IR. We conclude that *tie*-dependent increase in *ban* activity after IR was due, at least in part, to a *tie*-dependent increase in *ban* levels.

### The protective effect of *ptc-GAL4*-induced death is sensitive to *tie* gene dosage

Using the *ptc4>dE2f1^RNAi^* system, we addressed the role of *tie* in the protective effect of dying cells. Wing discs from *tie* Df(3L)9028 heterozygotes showed an attenuation of both *ban* activation, as seen by changes in the GFP *ban* sensor (Fig. S7 and Table 1), and the protective effect of cell death in the *ptc* domain (Fig. 5L, Table 1). This defect was rescued by one copy of *UAS-banA* (Fig. 5N and Table 1). We conclude that *tie* was required to activate *ban* and to confer protection in response to *ptc-GAL4*-induced cell death. Haplo-insufficiency of *tie* in these experiments was consistent with the identification of *tie* as a dominant modifier of *ban* in our screen.

### Pvf1, potential ligand for Tie, is up-regulated by IR and contributes to the protection

The ligands for mammalian Tie-2 are Angiopoietin 1 and 2. Although *Drosophila* genome includes five predicted Ang homologs [31], there is no evidence for these being ligands for *Drosophila* Tie. Instead, there is evidence for a *Drosophila* PDGF/VGF-like protein, Pvf1, in border cell migration, a process in which Tie is known to function [23]. We found that mRNA for Pvf1, Pvf2 and CG10359, which encodes one of the putative Ang homologs, were induced at 2 h after IR in wing imaginal discs ([32]; Supplemental Table 1). In p53 mutants, where caspase activation and apoptosis after irradiation were reduced and delayed [29], these genes were no longer induced. An independent RNAseq analysis confirmed these results (our unpublished data). In these experiments, mRNA levels for *tie*, another Pvf homolog, Pvf3, and four remaining predicted Ang homologs were unchanged (Table S1 and data not shown). Analysis of Pvf1 and Pvf2 expression using enhancer trap lines corroborated these results; we found that both genes were induced in the *ptc* domain after expression of *ptc4>dE2f1^RNAi^* (Figure S8).

These data suggest that cell death induced Pvf1, Pvf2 and/or CG10359 to activate Tie. To address this possibility, we monitored the protective effect of *ptc4>dE2f1^RNAi^* but in Pvf1 and Pvf2 mutants and a chromosomal deficiency that deletes CG10359. The results with Pvf2 and CG10359 were negative (not shown), but a hemizygote of a Pvf1 allele, shown before to express no detectable mRNA or protein [33], showed reduced protection (Fig. 5P and Table 1).

## Discussion

We document a previously unknown phenomenon in wing imaginal discs of *Drosophila* larvae; dying cells protected nearby cells from death. We found that killing cells by any one of three methods __ *ptc-GAL4*-driven expression of dE2F1^RNAi^ or pro-apoptotic genes *hid* and *rpr*, exposure to ionizing radiation (IR) and clonal induction of Hid/Rpr *__* activated an anti-apoptotic microRNA, *bantam*. Death by *ptc-GAL4* or clonal expression of Hid/Rpr also made surviving cells more resistant to killing by IR. The protective effect was sensitive to *ban* gene dosage. We have named this phenomenon ‘Mahakali effect’, after the Hindu goddess of death who protects her followers. Mahakali effect differs from classical radiation ‘bystander effect’ in which byproducts from cell corpses make surviving cells more prone to death [18], [19], [20]. The Mahakali effect appears to operate in a non-cell-autonomous fashion. Disc-wide protection by *ptc4>Rpr* and *Hid/Rpr* that included even cells in the P compartment that did not express *ptc*, provides the strongest evidence for non-autonomy. This idea is supported by the finding that IR-induced caspase activation was reduced in cells outside Hid/Rpr flip-out clones.

A recent paper describes a non-autonomous induction of apoptosis by apoptotic cells [34]. We do not believe these results necessarily contradict what is reported here. Most of the experiments in the published work used undead cells kept alive by p35; Mahakali effect is seen without p35. Non-autonomous apoptosis was assayed at, typically, 3–4 days after induction of undead cells; we detect Mahakali effect 6 hr after cell death induction using similar death-inducing stimuli (Hid/Rpr). It would be interesting to see how long Mahakali effect persists and whether non-autonomous apoptosis, occurring at longer time points, also produces Mahakali effects of its own. Another recent paper describes tissue regeneration after massive cell ablation in wing discs [35]. It would also be interesting to see if the Mahakali effect operates among regenerating cells.

**Figure 9:**
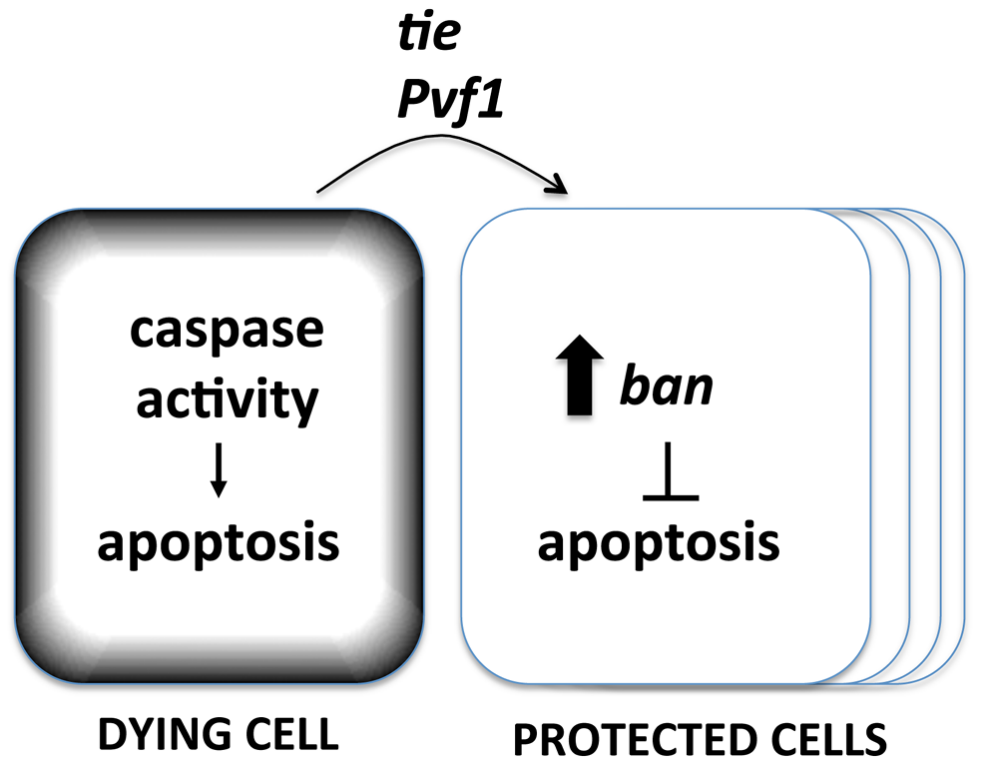
A model for the Mahakali effect. Known genetic determinants are shown.

The data shown here suggest that the basic components of the Mahakali effect are caspase activity in dying cells (because expression in dying cells of p35, an inhibitor of effector caspases, blocked *ban* activation), *ban* (because *ban* activation resulted from cell death and the protective effect was sensitive to *ban* gene dosage), and *tie* (because *tie* was required to activate *ban* and the protective effect was sensitive to *tie* gene dosage). We propose a model in which caspase activity in dying cells acts through Tie to cause non-autonomous activation of *ban* and the Mahakali effect (Figure 9). A validated target of *ban* in apoptosis inhibition is *hid,* whose 3′UTR includes 4 potential *ban* binding sites. We have shown previously that a GFP sensor with *hid* 3′UTR is reduced after IR [22], reflecting repression of *hid* by *ban*. Deletion of two potential *ban*-binding sites in the *hid* 3′UTR abolished the IR-induced changes in GFP.

The Mahakali effect differs in two ways from previously described effects of dead/dying cells in wing discs. First, the Mahakali effect extended further than previously reported signaling from dead/dying cells. In the extreme case of *ptc4>Hid/Rpr*, the protection reached as far as the edge of the disc. This distance, on of order of 100 or more mm (scale bar in Fig. 3) is comparable to the distance of border cell migration [36], in which Tie is known to function. In contrast, the mitogenic effect that occurs through JNK/Wingless in response to undead cells in the wing disc is seen up to 5 cells away [2]. Activation of proliferation through the Hpo/Yki axis also spans 3–5 cells away. This can be seen as activation of Yki targets such as DIAP1 [3]. We could reproduce this result: *ptc4>dE2f1^RNAi^* activated a Yki target, DIAP1, but only within or close to the *ptc* domain (Fig. S4). *Yki^B5^* allele, which disrupts cell death-induced proliferation [3], did not alter the Mahakali effect (Fig. S9), further supporting the idea that the two effects are different. Second, *ban* activation in response to cell death was sensitive to the caspase inhibitor p35. In contrast, the mitogenic effect of dying cells in wing imaginal discs is not sensitive to p35 [2], [37], [38], [39], [40], [41]. We note that the mitogenic effect of dying cells is inhibited by p35 in the differentiating posterior region of eye imaginal discs [42], which is similar to what we saw for *ban* activation in the wing discs.

We found that *tie* was required for IR-induced activation of *ban* and for larval survival after irradiation. There were similarities as well as differences in the role of *ban* and *tie*. *tie* mutants were IR-sensitive (this study), as are viable alleles of *ban* [22]. Tissue-specific overexpression of *ban* results in abnormal growth [5]; we found that 6 independent UAS-*tie* transgenic lines were lethal when driven by actin-GAL4 (data not shown). Thus, too much *ban* or *tie* has consequences. On the other hand, reducing *tie* or *ban* gene dosage by half attenuated the Mahakali effect. Thus, too little *ban* or *tie* also has consequences. In fact, UAS-*ban* or UAS-*tie* without a GAL4-driver was sufficient to rescue *ban* and *tie* mutant phenotypes [[6] and this study]. Thus, intermediate levels of expression may be important for the function of these genes.

The biggest difference between *ban* and *tie*, of course, was that while *tie* homozygous larvae were viable (this study), *ban* homozygous larvae are lethal [6]. *tie* became necessary only after radiation exposure. This suggests that *tie* was needed to regulate *ban* not during normal development but after radiation exposure. How is IR and cell death linked to Tie? We found that mRNA for Pvf1, a ligand for Tie in border cell migration, was induced by IR and that this induction appeared to be dependent on cell death (abolished in p53 mutants). *Pvf1^EP1624^* mutants that are mRNA and protein null [33], also showed reduced Mahakali effect. The degree of reduction was significant but not back to the level seen in control discs without *ptc4>dE2f1^RNAi^*, suggesting the involvement of additional ligands or mechanisms for Tie activation. In agreement, we could not see *ban* activation or the Mahakali effect after overproduction of Pvf1 (data not shown). Pvf1 was necessary but insufficient to produce these effects without cell death.

Tie activated *ban*, at least in part by increasing *ban* levels. How IR and caspase activity promotes Pvf1 expression and how Tie activity increases *ban* levels will be key questions to address in the future. Testing the role of known apoptosis regulators, such as Diap1, and signaling molecules, such as Wg, may help address these questions. We also plan to complete the genetic screen that identified Tie; it has the potential to identify additional components of the Mahakali effect.

Pvr, a PDGF/VEGF receptor homolog that function redundantly with Tie in border cell migration, also plays an anti-apoptotic role in embryonic hemocytes [43]. A recent study in wing discs found that Pvr is activated in neighbors of dying cells in a JNK-dependent manner, to result in cytoskeletal changes that allow the engulfment of the dead cell by the neighbor [44]. It is interesting that two PDGF/VEGF receptor homologs that function redundantly in cell migration during oogenesis may also play non-redundant roles in non-autonomous responses to cell death in wing discs.

Cancer therapy routinely comprises the application of two or more cytotoxic agents (taxol and radiation, for example) to cancer cells. A phenomenon in which cell killing by one agent influence resistance to the second agent is, therefore, of potential clinical significance. The bulk of our analysis focused on protection from IR-induced cell death. But we also have preliminary indication that the Mahakali effect can also protect against cell death induced by maytansinol (Fig. S10), a microtubule depolymerizing agent with relevance to cancer therapy that we found before to induce cell death in *Drosophila* wing discs [45]. An important question is whether a phenomenon like Mahakali effect exists in mammals and acts as a survival mechanism in response to cell death. Ang-1, a ligand for mammalian Tie-2, is a pro-survival factor for endothelial cells during serum deprivation and after irradiation in cell culture models [46], [47], [48]. Interestingly, Ang1 is produced not by endothelial cells but by neighbors, at least in cell culture [49]. Based on these data, we think it possible that radiation exposure results in Ang1 production by dead/dying cells that promote the survival of endothelial cells via Tie-2. Consistent, an Ang-1 derivative that is a potent activator of Tie-2 has been shown to protect endothelial cells from radiation-induced apoptosis [50].

## Materials and Methods

### Fly stocks

*ban* mutants: *ban* ^1170^, *ban* ^EP3622^, *ban* ^D1^ [5].

*tie* mutants:*y^1^ w^1118^; PBac{3HPy+}Tie^C098^* (Bloomington #16280); *PBAC{RB}Tie^e03394^* (Harvard Exelixis Collection); *PBAC{RB}Tie^e02680^* (Harvard Exelixis Collection); *w^1118^; Df(3L)Exel9028, PBac{RB5.WH5}Exel9028* (Bloomington #7925).

Pvf1 mutant: *pvf1^EP1624^* [33].

Clonal induction of GAL4: *hs-FLP^22^; 20.X/T(2;3) CyO Tb/Act<FRT>GAL4>UAS-RFP;* Generated using w^1118^; P{GAL4-Act5C(FRT.CD2).P}S, P{UAS-RFP.W}3/TM3, Sb^1^ (Bloomington#30558) and crossed to UAS-*hid*, *rpr* on Chr I [51].

Mitotic clones: *FRT80B-Df(3L)Exel9028 crossed to FRT80B-Ubi-GFP*

Other stocks: p*tc-GAL4* and UAS-dsRNA against dE2F1 or ‘PE3’ on Chr II [25]; *ban* sensor ‘20.X’ on Chr II [6]; Dpp-lac Z reporter, *P{BS3.0}H1-1, cn^1^; ry^506^* (Bloomington#5527); DIAP1-lac Z reporter [52]; Yki^5B^ [53]; UAS-ATM^RNAi^ on Chr III (VDRC stock #22502); Ptub-GAL80^ts^ on Chr III (Bloomington stock center); Pvf1 enhancer trap, w^1118^ Mi{ET1}Pvf1^MB01242^ (Bloomington#23032); Pvf2 enhancer trap, w^1118^ Mi{ET1}Pvf2^MB03230^ (Bloomington#24055).

### *tie* rescue construct

*To clone a Tie cDNA, total* RNA was isolated from whole *Sevelin* pupae 2-3 days post-white pupa stage, reverse transcribed (RT primer: ATGCGCTGCACGCCTAAATCA3), PCR-amplified with *tie* specific primers (amplification primers:5′ CGTGTGTGTATGTGTGTGTCG and

3′ GGGTAGGGGTTGGCTCAGTCA), and cloned into a pCR-XL-Topo vector (Invitrogen). Tie cDNA was sub-cloned into pUAST and injected into *w^1118^* embryos to make transgenic stocks at a commercial facility (Best Gene, Inc.). The integrity of the cDNA was verified by DNA sequence analysis after each cloning steps. Eight independent lines on Chromosome II and III were obtained. The data shown is with one Ch II line.

### Irradiation

Larvae in food were irradiated in a Faxitron Cabinet X-ray System Model RX-650 (Lincolnshire, IL) at 115 kv and 5.33 rad/sec. Irradiated larvae were incubated at 25°C for indicated amounts of time before dissection.

### Antibody, TUNEL, X-gal and Acridine Orange staining

Cleaved Caspase 3 (1∶100, rabbit polyclonal, Cell Signaling Cat# 3661) was used as described before [29]. Phospho-Histone H3 (Upstate Biotech, 1∶1000) and Engrailed (1∶500, Developmental Hybridoma Bank Cat#4D9) were used as described before [54]. Secondary antibodies were used at 1∶500 (Jackson). For TUNEL, wing discs were dissected in PBS, fixed in 4% para-formaldehyde in PBS for 20 minutes, and washed three times in 0.3% Triton X-100/PBS for at least 20 minutes total. The discs were permeabilized overnight at 4°C in 0.3% Triton X-100/PBS, followed by three washes in 0.3% Triton X-100/PBS for at least 10 minutes total. The discs were processed using an Apoptag Red kit (Millipore), according to manufacturer's instructions. For X-gal staining, wing discs were extirpated in PBS and fixed in PBS +4% formaldehyde for 10 min. The discs were washed three times for 5 min each in PBT (PBS +0.2% Tween 20). The discs were stained overnight in 1 mg/ml X-gal in staining solution (50 ml contains: 0.684 ml 1 M Na_2_HPO_4_, 0.316 ml 1 M Na_2_PO_4_, 1.5 ml 5 M NaCl, 0.05 ml 1 M MgCl_2_, 0.065 g K_4_Fe(II)(CN)_6_, 0.051 g K_3_Fe(II)(CN)_6_, 0.15 ml Triton X 100, 47 ml H_2_0). The discs were washed in PBT. The discs were counter-stained with 10 ug/ml Hoechst33258 in PBT or PBTx (0.1%TritonX-100) for 2 min, washed 3 times, and mounted on glass slides in Fluoromount G (SouthernBiotech).

Acridine Orange staining was as described before [29]. AO is excluded from live cells and has been shown to specifically stain apoptosis but not necrotic cells in *Drosophila* [55].

### Image analysis

To quantify the GFP sensor, wing imaginal discs were extirpated in PBS, mounted between a glass slide and a glass coverslip, and imaged live. For mean GFP signal per disc, the images were acquired on a Leica DMR fluorescence microscope using Slidebook (Intelligence Imagine), and compiled in Photoshop. For relative GFP signal between different parts of the same disc, images were acquired using a Perkin Elmers spinning disc confocal on a Leica DMR microscope. 26–36 Z sections 1 mm apart were collected and collapsed by maximal projection using Image J (NIH opensource), and mean GFP signal for defined areas measured. Anterior signals were normalized by dividing with the posterior signals and averaged for each sample.

To quantify caspase and TUNEL, discs were imaged on a Perkin Elmers spinning disc confocal attached to a Leica DMR microscope. 26–36 Z-sections 1 mm apart were collected per disc and collapsed using ‘maximum projection’ in Image J. Collapsed images were corrected for background using the ‘threshold’ function in Image J. The mean fluorescence from the area of interest was measured and averaged for all discs in a sample. To quantify mitotic indices, cells showing phosphor-Histone H3 stain were manually counted.

### Quantitative RT-PCR

Larvae were irradiated at 96±2 hr AEL. Between 15–20 wing discs were dissected per sample per time point and flash frozen in PBS using liquid nitrogen. Total RNA was isolated using Invitrogen TRIzol kit according to the manufacturer's instructions, and treated with DNase I (Amplification Grade, Invitrogen). RT reactions were performed with Superscript III (Invitrogen) according to the manufacturers instructions, using primers to amplify NT1, *tie,* CG11353 or a-tubulin mRNA as control. The primers used were:

*tie:*

5′ GGCGACGGGAAAGCCGAAA

3′ GGTGCGACGAGCAGCCAACA

NT1:

5′ GGCGGATGAGGGATTGCGCC

3′ TGCCAAACATCATGCGAACCTGT

CG11353:

5′ AGCGCGGCATACTCGGCAAA

3′ GGTCTTTGGACGCCGCGACA

a-Tub84B (a-tubulin):

5′TCCAATCGCAACAAAAAATTCA

3′ TCGTTTTCGTATGCTTTTCAGTGT

For mature *bantam* miRNA, custom primers were purchased and used according to manufacturer's instructions (TaqMan, MicroRNA Assay, Applied Biosystems).

Q-PCR was performed using 1 X SYBR Green Mix (Applied Biosystems) and 4 ng of each cDNA for 35 cycles using the indicated primers. Standard curves using 0.01-20 ng of cDNA pools were used. Plates were read in an Applied Biosystems 7900HT Real-time PCR instrument (Absolute Quantification Method). Values were normalized to those of a-tubulin.

### Statistical analysis

p-values were calculated using 2-tailed Student's t-test except in the screen that used X^2^ tests.

## Supporting Information

Figure S1 Activation of *ban* after induction of cell death in wing discs. (relates to Figure 1). (A, B) Wing discs were isolated 4 hours (h) after exposure to 0 R (-IR) or 4000 R (+IR) of X-rays, fixed and stained for cleaved Caspase 3. (C–F) GFP images of wing discs from larva carrying the *ban* sensor (C, D) or the control sensor that lacked *ban* binding sites (E, F). Wing discs were extirpated and imaged live from larvae 24 h after irradiation with 0 R (-IR) or 4000 R (+IR) of X-rays. (G) Mean GFP signal for each disc was quantified from images such as those in (C–F) and normalized to –IR values. ‘ctrl’  =  control sensor. (H, I) Wing discs with clones expressing RFP (H) or RFP, Hid/Rpr, and caspase inhibitor p35 (I) were imaged live 48 h after heat-shock induction of GAL4. Larvae were heat-shocked at 37°C for 10 min at 2 days after egg collection. ‘RFP’  =  *hs-FLP/Y;* GFP ban sensor*/+;UAS-p35/Act>>GAL4, UAS-RFP*. ‘RFP, Hid/Rpr,p35’ carried the same transgenes and had *UAS-hid, UAS-rpr* instead of Y.
(PDF)

Figure S2 Maturation of the *ptc* domain and comparison of cell killing due to E2F1 RNAi and ATM RNAi. (relates to Figures 2 and 3). (A–C) Wing discs from larvae carrying one copy each of *ptc-GAL4*, UAS-dsRNA against dE2F1 and UAS-GFP, at 72 h (A) or 96 h (B, C) after egg deposition (AED) at 25°C. The *ptc* strip did not span the wing pouch at 72 h AED and narrowed further by 96 h AED. The larva in (C) carried a copy of *tub-GAL80^ts^* that repressed GAL4 and GFP expression at this temperature. Scale bar in (C) applies to (A–C). (D–F) Wing discs were extirpated from third instar larvae and stained with the vital dye acridine orange. (D) A wing disc from a *ptc4>dE2F1^RNAi^* larvae raised at 25°C before shifting to 29°C for 24 h. Robust cell death was apparent at this time after temperature shift. (E, F) Wing disc from larvae expressing dsRNA against ATM under the control of *ptc-GAL4*. Larvae were raised at 25°C before shifting to 29°C for 24 h (e) or 48 h (D). Cell death was induced only after longer temperature shift and, even then, was not as robust as in *ptc4>dE2F1^RNAi^* discs. Scale bar in (F) applies to (D–F).
(PDF)

Figure S3 Mitotic Indices in anterior and posterior compartments are similar. (relates to Figure 3). Wing imaginal discs were fixed and stained for DNA (A, B) and for phosphorylated histone H3 (pH3) as a mitotic marker (C, D). DNA stain was used as a guide to circle the pouch and to mark the Anterior/Posterior boundary. Mitotic index was computed by manually counting pH3-positive cells and normalizing by the area measured using Image J. Mitotic index of the Anterior was divided by the mitotic index of the Posterior compartment for each disc and shown in the graph in (E). N = 8 in two experiments for CyO discs. The averages are indicated with horizontal bars for each sample. The numbers are not significantly different from each other (p = 0.37).
(PDF)

Figure S4 Expression of Dpp-lacZ and DIAP1-lacZ reporters in discs with cell death (relates to Figure 3). Wing discs were extirpated from feeding third instar larvae and stained to detect β-galactocidase. Larvae were maintained at 25°C for 4 days and shifted to 29°C for 24 h before dissection. Larvae carried either the CyO balancer (A and C) or transgenes for *ptc-GAL4* and UAS-dsRNA against dE2F1 (B and D). The larvae also carried a Dpp-lacZ reporter (A and B) or a DIAP1-lacZ reporter (C and D). The stripe of Dpp-lacZ expression remained even in discs in which some cells had been killed in the *ptc* domain (B), and looked similar to Dpp-lacZ expression in CyO controls (A). *ptc4>dE2F1^RNAi^* induced the expression of DIAP1 (D) compared to CyO controls (C). Note that induction of DIAP1 was confined to within or proximity of the *ptc* domain and did not spread to the entire anterior compartment of the pouch.
(PDF)

Figure S5 A screen for modifiers of radiation sensitivity of *ban* mutants (relates to Figure 7). (A) A screen for modifiers of *ban* was designed to identify deficiencies that dominantly modulated the radiation sensitivity of *ban* mutants. Larvae were exposed to 4000 R of X-rays at 96±4 h after egg deposition. Percent eclosion was determined by counting full and empty pupal cases 10 days after irradiation. To calculate the expected survival from additive effects of *ban* and the deficiency (Df), *ban^L1170^/*TM6 Tb or *ban^EP3622^/*TM6 Tb virgin females were crossed to +/+ males and Df/TM6 Tb males were crossed to +/+ virgin females. Percent eclosions for Tb+ pupae (*ban*/+ and Df/+) were multiplied with each other to get the expected survival. For example, if % eclosion for *ban*/+ and Df/+ were 60% and 60%, the expected survival would be 0.6×0.6 or 36%. To measure the observed survival, *ban/*TM6 Tb virgin females were crossed to Df/TM6 Tb males. X^2^ analysis (1 df) was used to calculate the significance of the difference between observed and expected survival, using a cut of value of p<0.001. (B) *tie* locus on 3R is shown to scale. Predicted and known genes are blue bars. NT1 encodes Neurotrophin 1, which has a role in axonal activity. *tie* transcript is in orange; boxes are exons and lines are introns. Transposon insertion sites for three *tie* alleles used in this study are indicated with red triangles. *tie^e03394^* and *tie^e02680^* are p-element insertions into the second intron and *tie^CO98^* is a p-element insertion into the predicted 3′UTR. The deficiency used in this study removes the region shown as a red bar. All information are from Flybase (FB2012_02, released 03/02/12). (C) mRNA expression levels of *tie* and two flanking genes, CG11353 and NT1, in wild type and *tie* mutants were quantified by q-RT-PCR. The average and standard error from three independent experiments are shown. The data have been normalized to expression in wild type (*w^1118^* or ‘w’). *tie* alleles were Df(3L)Exel2098 and *tie^e03394^*.
(PDF)

Figure S6 *tie* mutants can induce cell death after irradiation (relates to Figure 8). (A–C) Wing imaginal discs were extirpated from third instar larvae at 4 or 20 h after exposure to 4000R of X-rays, fixed and stained with an antibody against cleaved (active) Caspase 3 and for DNA. To image discs, Z-sections were acquired on a spinning disc confocal microscope, collapsed and shown. Representative images from the 4 h time point are shown. (D–E) Mean fluorescence for each disc was measured using Image J software and normalized to the average mean fluorescence of *w^1118^* control discs. ‘Df’  =  homozygotes of Df(3L)Exel2098; ‘e03394’  =  *tie^e03394^* homozygotes. Error bar =  ±1SEM. N = 15–23 discs per genotype for each time point in two independent experiments. (F–I) Homozygous mutant clones of *tie* Df(3L)Exel2098 (no GFP) showed robust caspase activity 4 h and 24 h after exposure to 4000R of X-rays. Larvae were irradiated 48 h after heat shock to induce FLP recombinase. Homozygous mutant clones lack GFP, heterozygotes have one copy of GFP and homozygous wild type sister clones have two copies of GFP.
(PDF)

Figure S7 Changes in the GFP *ban* sensor is sensitive to *tie* gene dosage (relates to Table 1). To image discs, Z-sections were acquired on a spinning disc confocal microscope, collapsed and shown. Brackets indicate bright GFP along the D/V boundary. (A) A control disc from a larva carrying the GFP *ban* sensor that had been subjected to the experimental protocol in (C). GFP from the *ban* sensor was equivalent in the A (black arrowheads) and the P (white arrowheads) compartments of the wing pouch. (B) In *tie Df*/+ discs with cell death due to *ptc4>dE2F1^RNAi^*, the reduction of GFP in the A compartment was not obvious (A/P ratio quantified in Table 1). A few speckles of dying cells with bright GFP were visible in the *ptc* domain (* in B).
(PDF)

Figure S8 The expression of Pvf1 and Pvf2 enhancer traps is induced by *ptc4>dE2F1^RNAi^* (relates to Figure 5). Wing imaginal discs were extirpated from third instar larvae 24 h after a temperature shift to 29°C, and imaged live for GFP. The larvae carried GFP enhancer traps for Pvf1 (A, B) and Pvf2 (C, D). The larvae were heterozygous for the CyO-RFP balancer (A, C) or *ptc4>dE2F1^RNAi^* (B, D). Induction of GFP from the enhancer traps was observed in the *ptc* domain where the dying cells are. N = 10 for each genotype/panel in two independent experiments.
(PDF)

Figure S9 Yki heterozygotes still show protection from IR-induced cell death in the *ptc4>dE2F1^RNAi^* background (relates to Figures 3 & 4). Wing imaginal discs were extirpated from third instar larvae 4 h after exposure to 4000R of X-rays, fixed and stained for DNA and with an antibody against cleaved Caspase 3. The larvae are heterozygous for *ptc4>dE2F1^RNAi^* and *yki^B5^*. DNA stained images are used to discern the location of the pouch (within the dashed line), anterior/posterior boundary (solid vertical line) and the *ptc* domain (dotted vertical line). The horizontal stripe of cell death along the dorsal/ventral boundary is also seen here. Importantly, caspase-active cells are fewer in the anterior half than in the posterior half.
(PDF)

Figure S10 Cell death in the *ptc* domain protects against maytansinol. Larvae were generated to express *ptc-GAL4>UAS-Rpr* as shown in Figure 3K. Immediately after de-repressing GAL4 for 12 h, maytansinol (NSC292222, Developmental Therapeutics Program, NCI) in DMSO was added to the food to a final concentration of 2 μM (and 0.1% DMSO). Wing discs were extirpated 24 hours after drug addition, fixed and stained for DNA and active Caspse 3. (A, B) male siblings that lack the *UAS-rpr* transgene served as controls. Cell death in response to maytansinol was equivalent in the A and P compartments. (C, D) female experimental larvae showed cell death in the *ptc* domain (between vertical lines). Caspase activity in the A compartment was lower than in the P compartment. We note that the depression in the DNA stain in the *ptc* domain was not visible in these discs. Maytansinol depolymerizes microtubule, suggesting that microtubules are necessary for the protrusion of dead cells.
(PDF)

Table S1 Changes in mRNA levels at 2 hours after IR. The data for *pvf1*, *pvf2* and *CG10359* are from published supplemental microarray data in Ref#30. The data for *tie* and *pvf3* are from an RNAseq dataset (our unpublished data).
(PDF)
