# Supplementary figures and images for: Dying Cells Protect Survivors from Radiation-Induced Cell Death in Drosophila
Source: PLoS Genet. 2014 Mar 27;10(3):e1004220. doi: 10.1371/journal.pgen.1004220 (PMC3967929; doi:10.1371/journal.pgen.1004220)

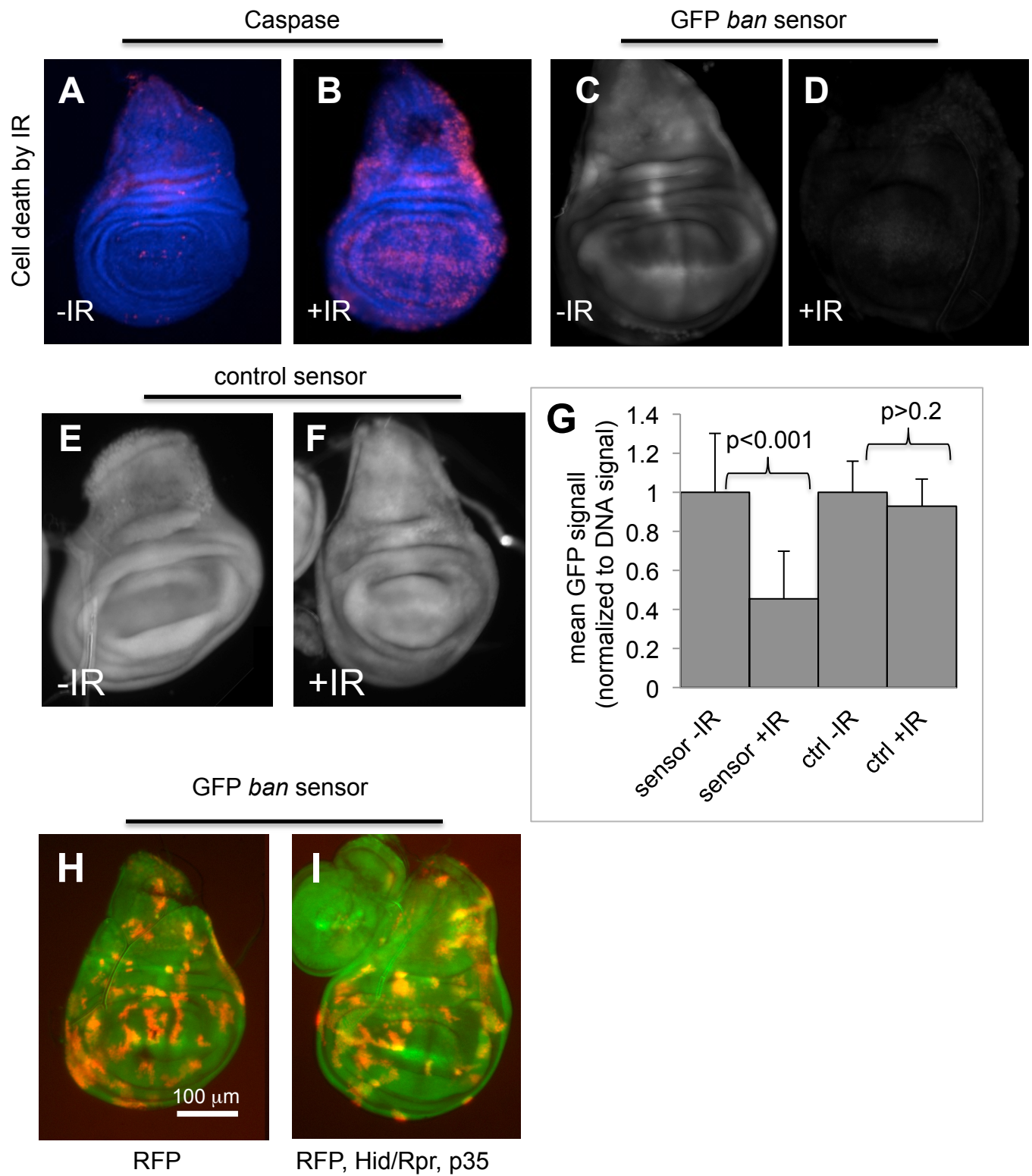

Supplement: Figure S1 — Activation of ban after induction of cell death in wing discs. (relates to Figure 1). (A, B) Wing discs were isolated 4 hours (h) after exposure to 0 R (-IR) or 4000 R (+IR) of X-rays, fixed and stained for cleaved Caspase 3. (C–F) GFP images of wing discs from larva carrying the ban sensor (C, D) or the control sensor that lacked ban binding sites (E, F). Wing discs were extirpated and imaged live from larvae 24 h after irradiation with 0 R (-IR) or 4000 R (+IR) of X-rays. (G) Mean GFP signal for each disc was quantified from images such as those in (C–F) and normalized to –IR values. ‘ctrl’ = control sensor. (H, I) Wing discs with clones expressing RFP (H) or RFP, Hid/Rpr, and caspase inhibitor p35 (I) were imaged live 48 h after heat-shock induction of GAL4. Larvae were heat-shocked at 37°C for 10 min at 2 days after egg collection. ‘RFP’ = hs-FLP/Y; GFP ban sensor/+;UAS-p35/Act>>GAL4, UAS-RFP. ‘RFP, Hid/Rpr,p35’ carried the same transgenes and had UAS-hid, UAS-rpr instead of Y. (PDF) [file pgen.1004220.s001.pdf]

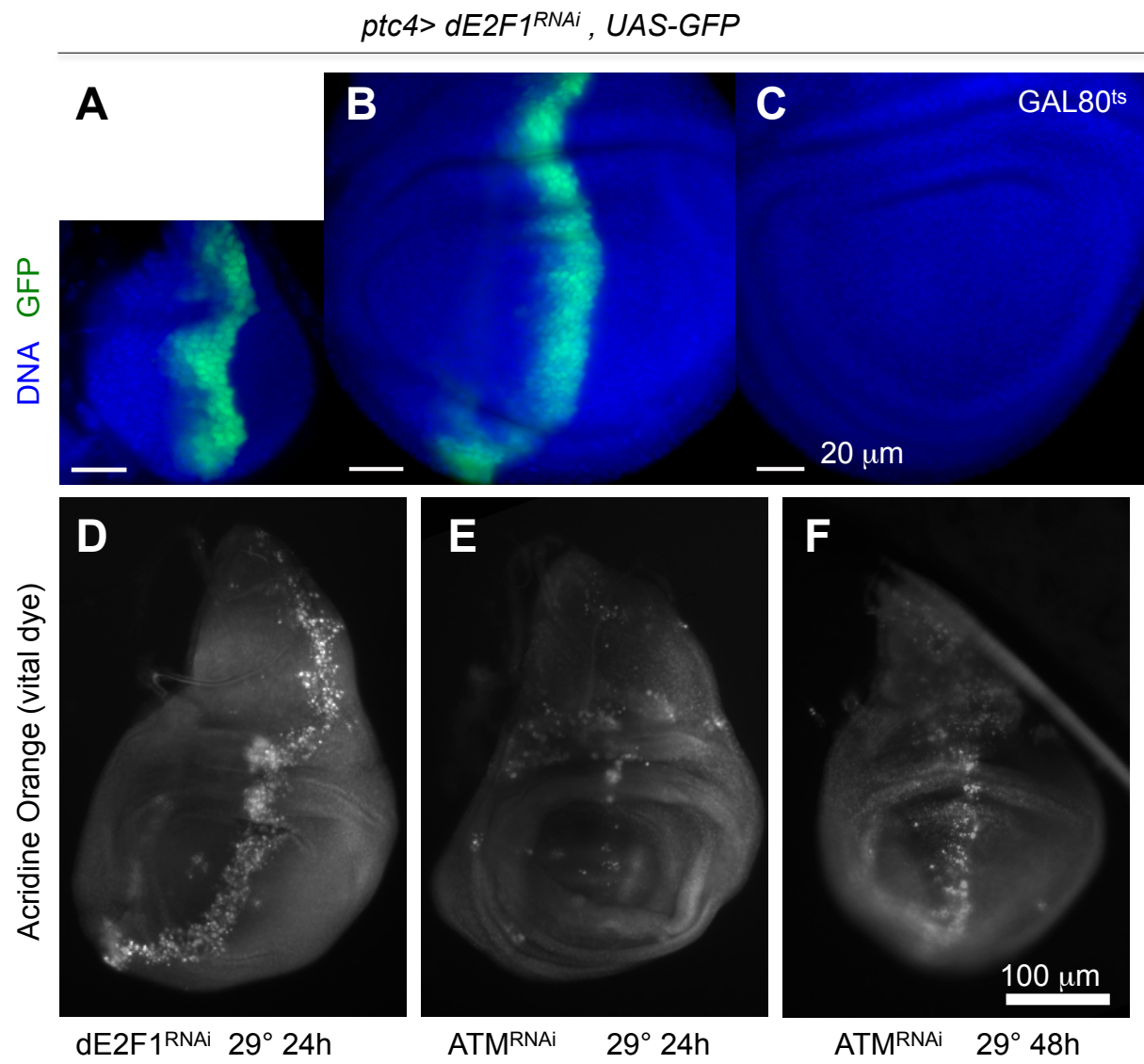

Supplement: Figure S2 — Maturation of the ptc domain and comparison of cell killing due to E2F1 RNAi and ATM RNAi. (relates to Figures 2 and 3). (A–C) Wing discs from larvae carrying one copy each of ptc-GAL4, UAS-dsRNA against dE2F1 and UAS-GFP, at 72 h (A) or 96 h (B, C) after egg deposition (AED) at 25°C. The ptc strip did not span the wing pouch at 72 h AED and narrowed further by 96 h AED. The larva in (C) carried a copy of tub-GAL80ts that repressed GAL4 and GFP expression at this temperature. Scale bar in (C) applies to (A–C). (D–F) Wing discs were extirpated from third instar larvae and stained with the vital dye acridine orange. (D) A wing disc from a ptc4>dE2F1RNAi larvae raised at 25°C before shifting to 29°C for 24 h. Robust cell death was apparent at this time after temperature shift. (E, F) Wing disc from larvae expressing dsRNA against ATM under the control of ptc-GAL4. Larvae were raised at 25°C before shifting to 29°C for 24 h (e) or 48 h (D). Cell death was induced only after longer temperature shift and, even then, was not as robust as in ptc4>dE2F1RNAi discs. Scale bar in (F) applies to (D–F). (PDF) [file pgen.1004220.s002.pdf]

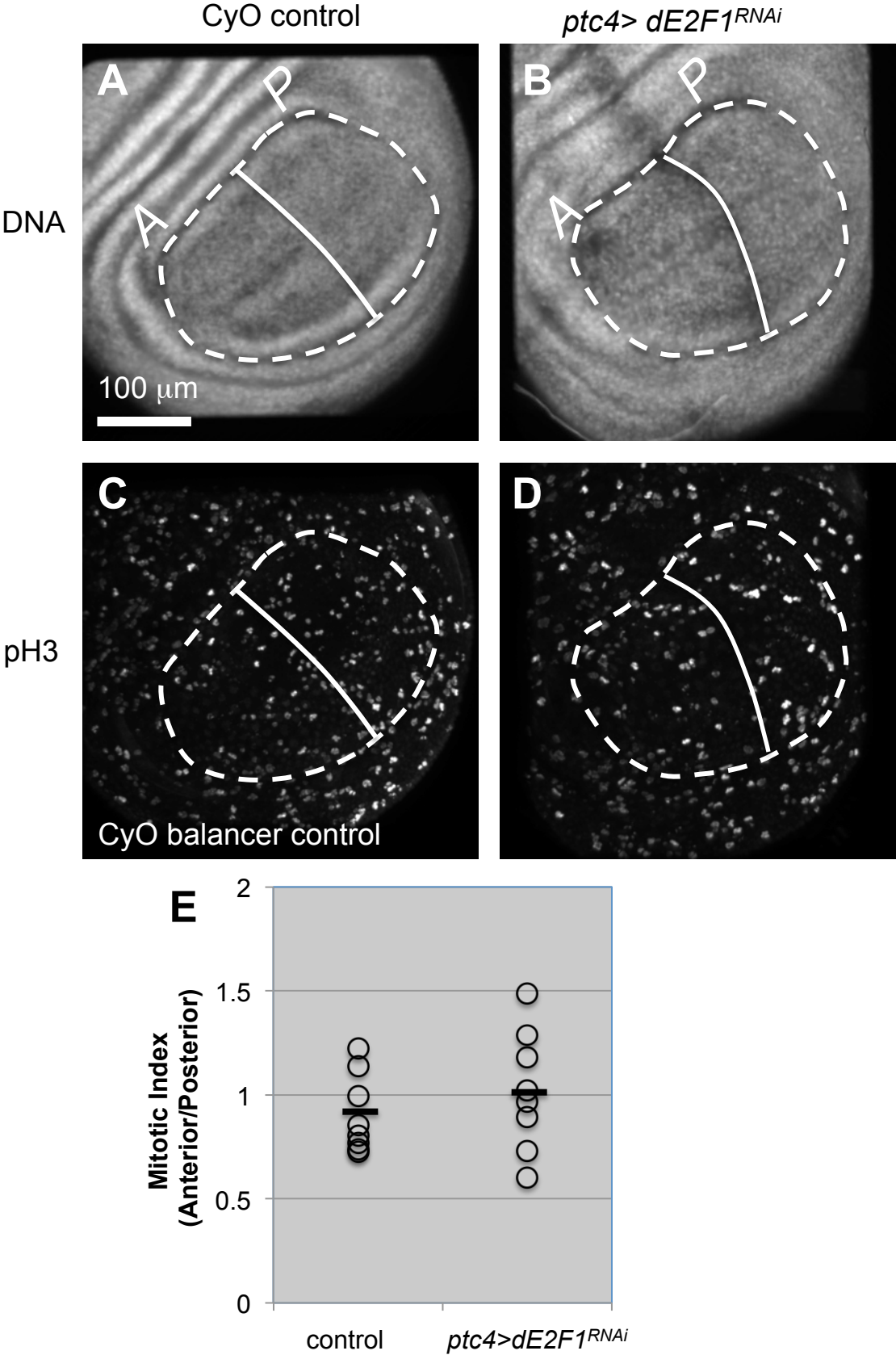

Supplement: Figure S3 — Mitotic Indices in anterior and posterior compartments are similar. (relates to Figure 3). Wing imaginal discs were fixed and stained for DNA (A, B) and for phosphorylated histone H3 (pH3) as a mitotic marker (C, D). DNA stain was used as a guide to circle the pouch and to mark the Anterior/Posterior boundary. Mitotic index was computed by manually counting pH3-positive cells and normalizing by the area measured using Image J. Mitotic index of the Anterior was divided by the mitotic index of the Posterior compartment for each disc and shown in the graph in (E). N = 8 in two experiments for CyO discs. The averages are indicated with horizontal bars for each sample. The numbers are not significantly different from each other (p = 0.37). (PDF) [file pgen.1004220.s003.pdf]

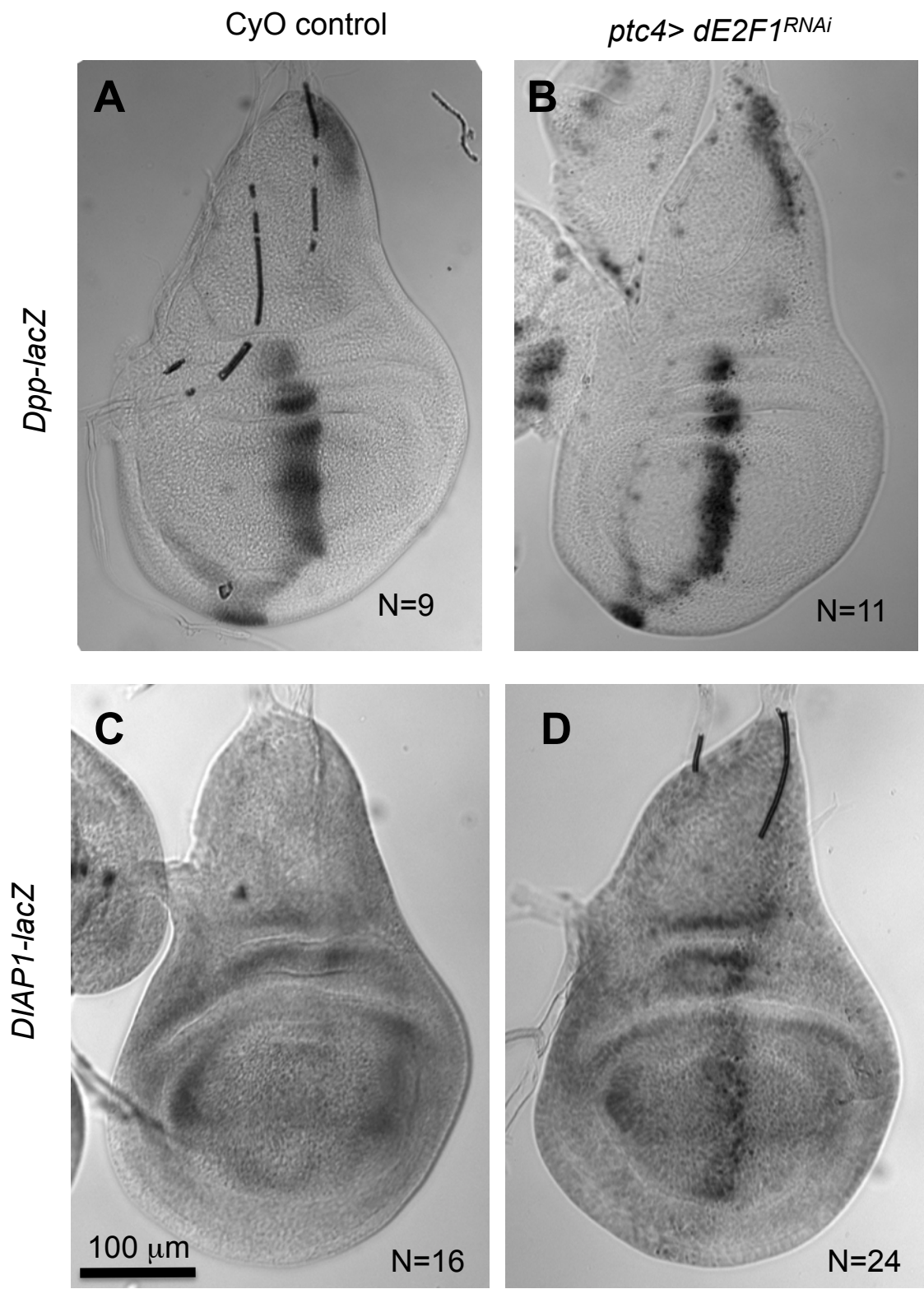

N=number of discs examined in two independent experiments

Supplement: Figure S4 — Expression of Dpp-lacZ and DIAP1-lacZ reporters in discs with cell death (relates to Figure 3). Wing discs were extirpated from feeding third instar larvae and stained to detect β-galactocidase. Larvae were maintained at 25°C for 4 days and shifted to 29°C for 24 h before dissection. Larvae carried either the CyO balancer (A and C) or transgenes for ptc-GAL4 and UAS-dsRNA against dE2F1 (B and D). The larvae also carried a Dpp-lacZ reporter (A and B) or a DIAP1-lacZ reporter (C and D). The stripe of Dpp-lacZ expression remained even in discs in which some cells had been killed in the ptc domain (B), and looked similar to Dpp-lacZ expression in CyO controls (A). ptc4>dE2F1RNAi induced the expression of DIAP1 (D) compared to CyO controls (C). Note that induction of DIAP1 was confined to within or proximity of the ptc domain and did not spread to the entire anterior compartment of the pouch. (PDF) [file pgen.1004220.s004.pdf]

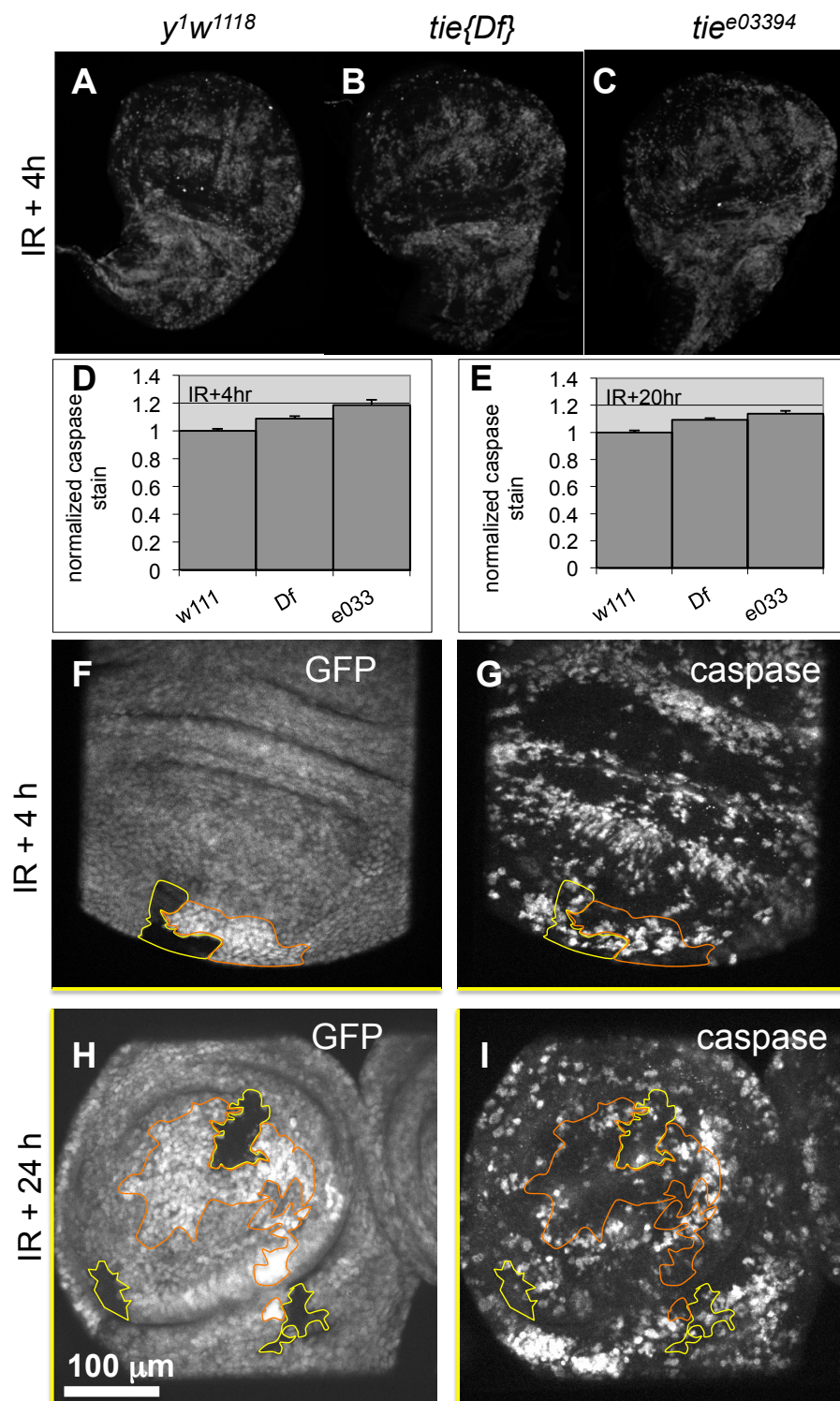

Supplement: Figure S6 — tie mutants can induce cell death after irradiation (relates to Figure 8). (A–C) Wing imaginal discs were extirpated from third instar larvae at 4 or 20 h after exposure to 4000R of X-rays, fixed and stained with an antibody against cleaved (active) Caspase 3 and for DNA. To image discs, Z-sections were acquired on a spinning disc confocal microscope, collapsed and shown. Representative images from the 4 h time point are shown. (D–E) Mean fluorescence for each disc was measured using Image J software and normalized to the average mean fluorescence of w1118 control discs. ‘Df’ = homozygotes of Df(3L)Exel2098; ‘e03394’ = tiee03394 homozygotes. Error bar = ±1SEM. N = 15–23 discs per genotype for each time point in two independent experiments. (F–I) Homozygous mutant clones of tie Df(3L)Exel2098 (no GFP) showed robust caspase activity 4 h and 24 h after exposure to 4000R of X-rays. Larvae were irradiated 48 h after heat shock to induce FLP recombinase. Homozygous mutant clones lack GFP, heterozygotes have one copy of GFP and homozygous wild type sister clones have two copies of GFP. (PDF) [file pgen.1004220.s006.pdf]

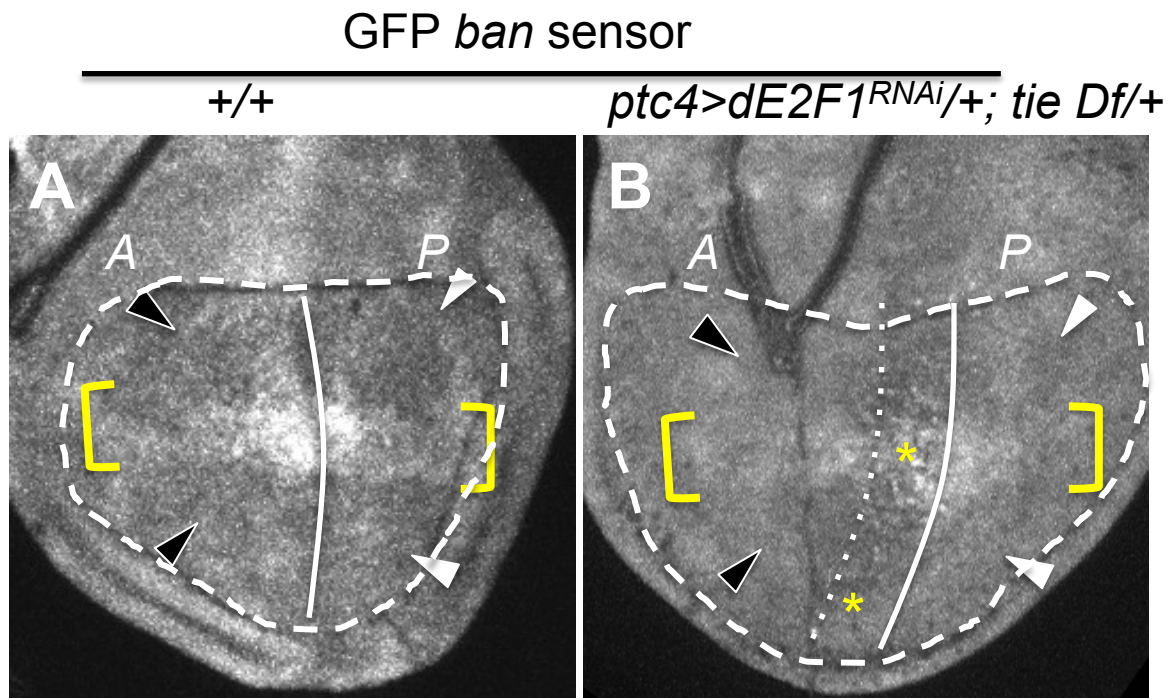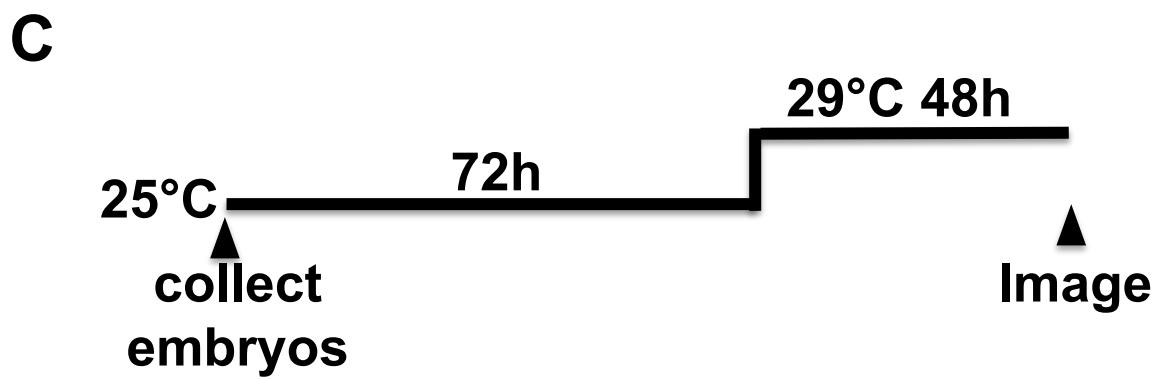

Supplement: Figure S7 — Changes in the GFP ban sensor is sensitive to tie gene dosage (relates to Table 1). To image discs, Z-sections were acquired on a spinning disc confocal microscope, collapsed and shown. Brackets indicate bright GFP along the D/V boundary. (A) A control disc from a larva carrying the GFP ban sensor that had been subjected to the experimental protocol in (C). GFP from the ban sensor was equivalent in the A (black arrowheads) and the P (white arrowheads) compartments of the wing pouch. (B) In tie Df/+ discs with cell death due to ptc4>dE2F1RNAi, the reduction of GFP in the A compartment was not obvious (A/P ratio quantified in Table 1). A few speckles of dying cells with bright GFP were visible in the ptc domain (* in B). (PDF) [file pgen.1004220.s007.pdf]

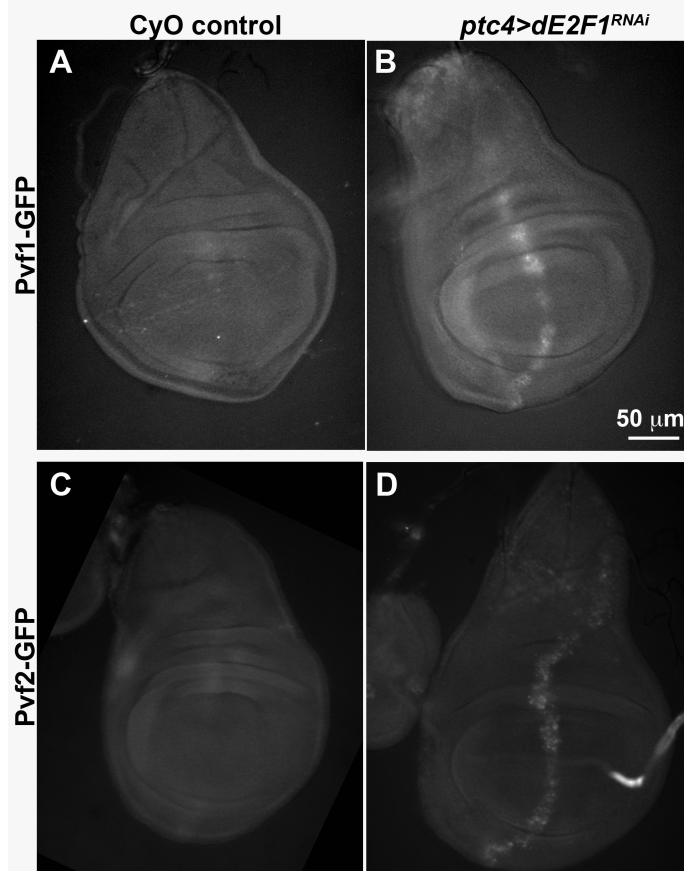

Supplement: Figure S8 — The expression of Pvf1 and Pvf2 enhancer traps is induced by ptc4>dE2F1RNAi (relates to Figure 5). Wing imaginal discs were extirpated from third instar larvae 24 h after a temperature shift to 29°C, and imaged live for GFP. The larvae carried GFP enhancer traps for Pvf1 (A, B) and Pvf2 (C, D). The larvae were heterozygous for the CyO-RFP balancer (A, C) or ptc4>dE2F1RNAi (B, D). Induction of GFP from the enhancer traps was observed in the ptc domain where the dying cells are. N = 10 for each genotype/panel in two independent experiments. (PDF) [file pgen.1004220.s008.pdf]

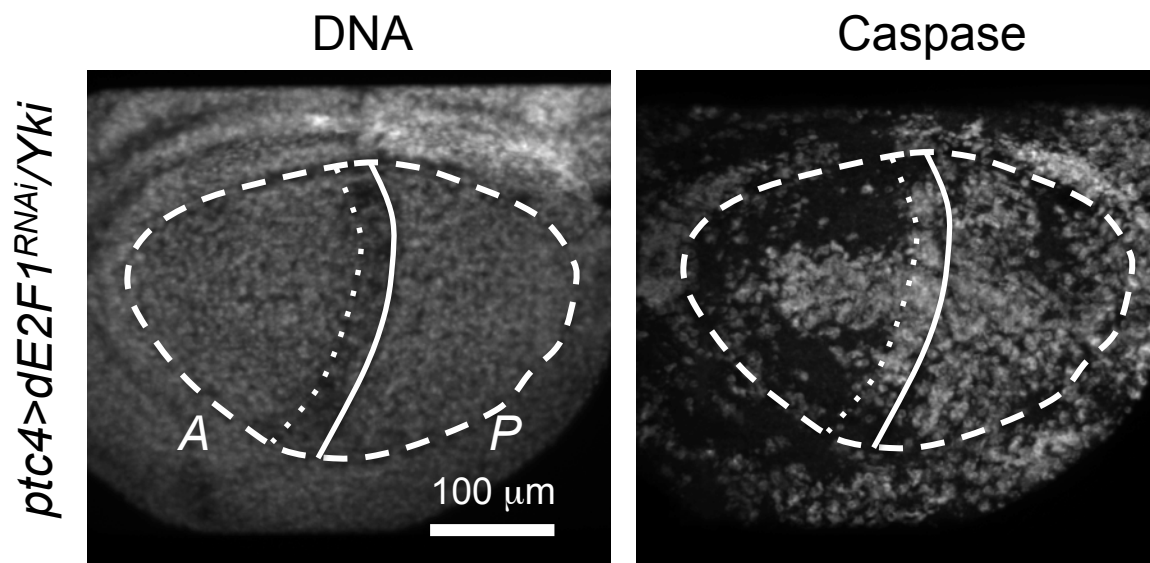

Supplement: Figure S9 — Yki heterozygotes still show protection from IR-induced cell death in the ptc4>dE2F1RNAi background (relates to Figures 3 & 4). Wing imaginal discs were extirpated from third instar larvae 4 h after exposure to 4000R of X-rays, fixed and stained for DNA and with an antibody against cleaved Caspase 3. The larvae are heterozygous for ptc4>dE2F1RNAi and ykiB5. DNA stained images are used to discern the location of the pouch (within the dashed line), anterior/posterior boundary (solid vertical line) and the ptc domain (dotted vertical line). The horizontal stripe of cell death along the dorsal/ventral boundary is also seen here. Importantly, caspase-active cells are fewer in the anterior half than in the posterior half. (PDF) [file pgen.1004220.s009.pdf]

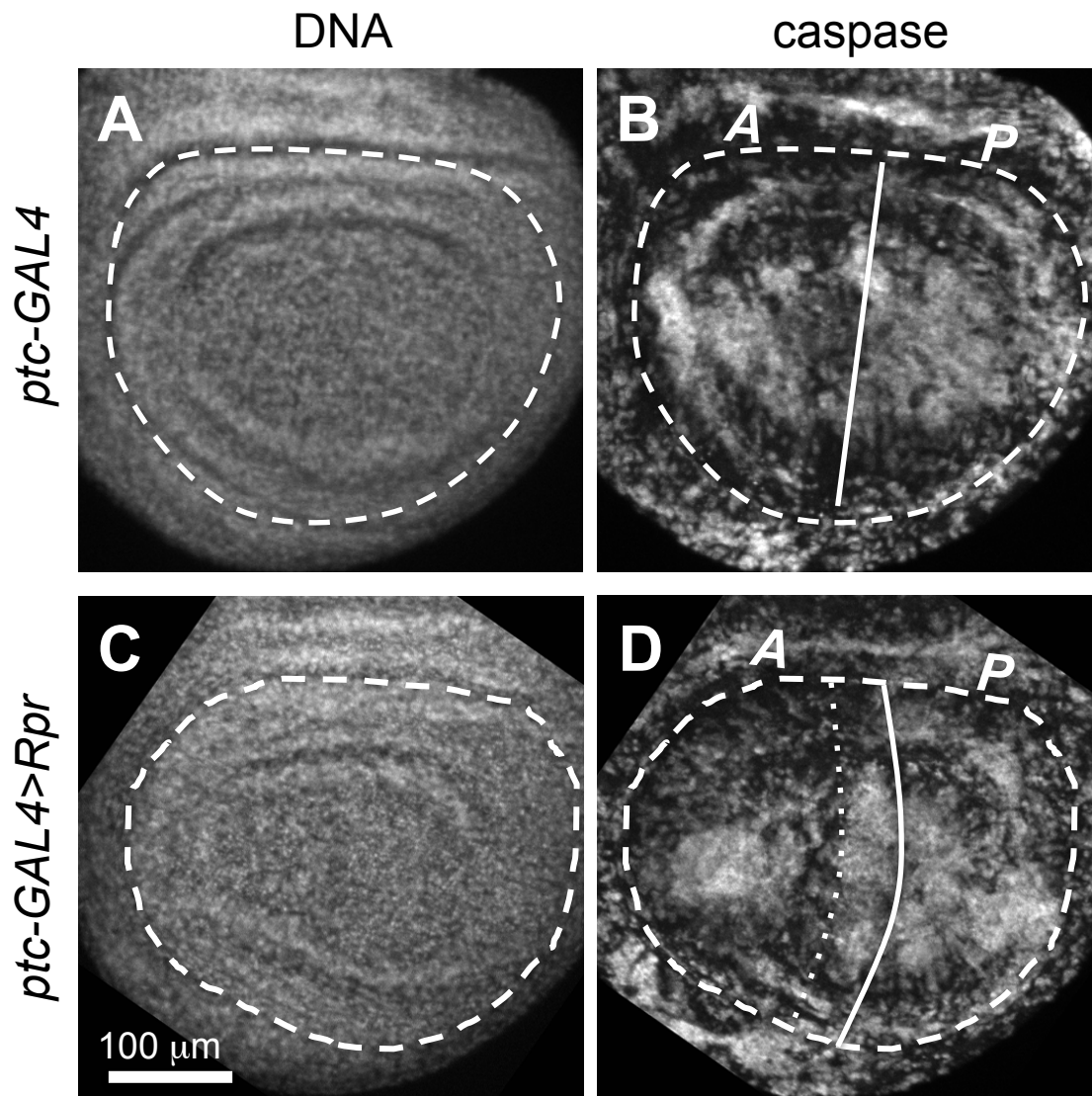

Supplement: Figure S10 — Cell death in the ptc domain protects against maytansinol. Larvae were generated to express ptc-GAL4>UAS-Rpr as shown in Figure 3K. Immediately after de-repressing GAL4 for 12 h, maytansinol (NSC292222, Developmental Therapeutics Program, NCI) in DMSO was added to the food to a final concentration of 2 μM (and 0.1% DMSO). Wing discs were extirpated 24 hours after drug addition, fixed and stained for DNA and active Caspse 3. (A, B) male siblings that lack the UAS-rpr transgene served as controls. Cell death in response to maytansinol was equivalent in the A and P compartments. (C, D) female experimental larvae showed cell death in the ptc domain (between vertical lines). Caspase activity in the A compartment was lower than in the P compartment. We note that the depression in the DNA stain in the ptc domain was not visible in these discs. Maytansinol depolymerizes microtubule, suggesting that microtubules are necessary for the protrusion of dead cells. (PDF) [file pgen.1004220.s010.pdf]
