# Supplementary material for: Dying Cells Protect Survivors from Radiation-Induced Cell Death in Drosophila
Source: PLoS Genet. 2014 Mar 27;10(3):e1004220. doi: 10.1371/journal.pgen.1004220 (PMC3967929; doi:10.1371/journal.pgen.1004220)
Supplement: Figure S5 — A screen for modifiers of radiation sensitivity of ban mutants (relates to Figure 7). (A) A screen for modifiers of ban was designed to identify deficiencies that dominantly modulated the radiation sensitivity of ban mutants. Larvae were exposed to 4000 R of X-rays at 96±4 h after egg deposition. Percent eclosion was determined by counting full and empty pupal cases 10 days after irradiation. To calculate the expected survival from additive effects of ban and the deficiency (Df), banL1170/TM6 Tb or banEP3622/TM6 Tb virgin females were crossed to +/+ males and Df/TM6 Tb males were crossed to +/+ virgin females. Percent eclosions for Tb+ pupae (ban/+ and Df/+) were multiplied with each other to get the expected survival. For example, if % eclosion for ban/+ and Df/+ were 60% and 60%, the expected survival would be 0.6×0.6 or 36%. To measure the observed survival, ban/TM6 Tb virgin females were crossed to Df/TM6 Tb males. X2 analysis (1 df) was used to calculate the significance of the difference between observed and expected survival, using a cut of value of p<0.001. (B) tie locus on 3R is shown to scale. Predicted and known genes are blue bars. NT1 encodes Neurotrophin 1, which has a role in axonal activity. tie transcript is in orange; boxes are exons and lines are introns. Transposon insertion sites for three tie alleles used in this study are indicated with red triangles. tiee03394 and tiee02680 are p-element insertions into the second intron and tieCO98 is a p-element insertion into the predicted 3′UTR. The deficiency used in this study removes the region shown as a red bar. All information are from Flybase (FB2012_02, released 03/02/12). (C) mRNA expression levels of tie and two flanking genes, CG11353 and NT1, in wild type and tie mutants were quantified by q-RT-PCR. The average and standard error from three independent experiments are shown. The data have been normalized to expression in wild type (w1118 or ‘w’). tie alleles were Df(3L)Exel2098 and tiee03394. [file pgen.1004220.s005.pdf]

Bilak et al. Figure S5

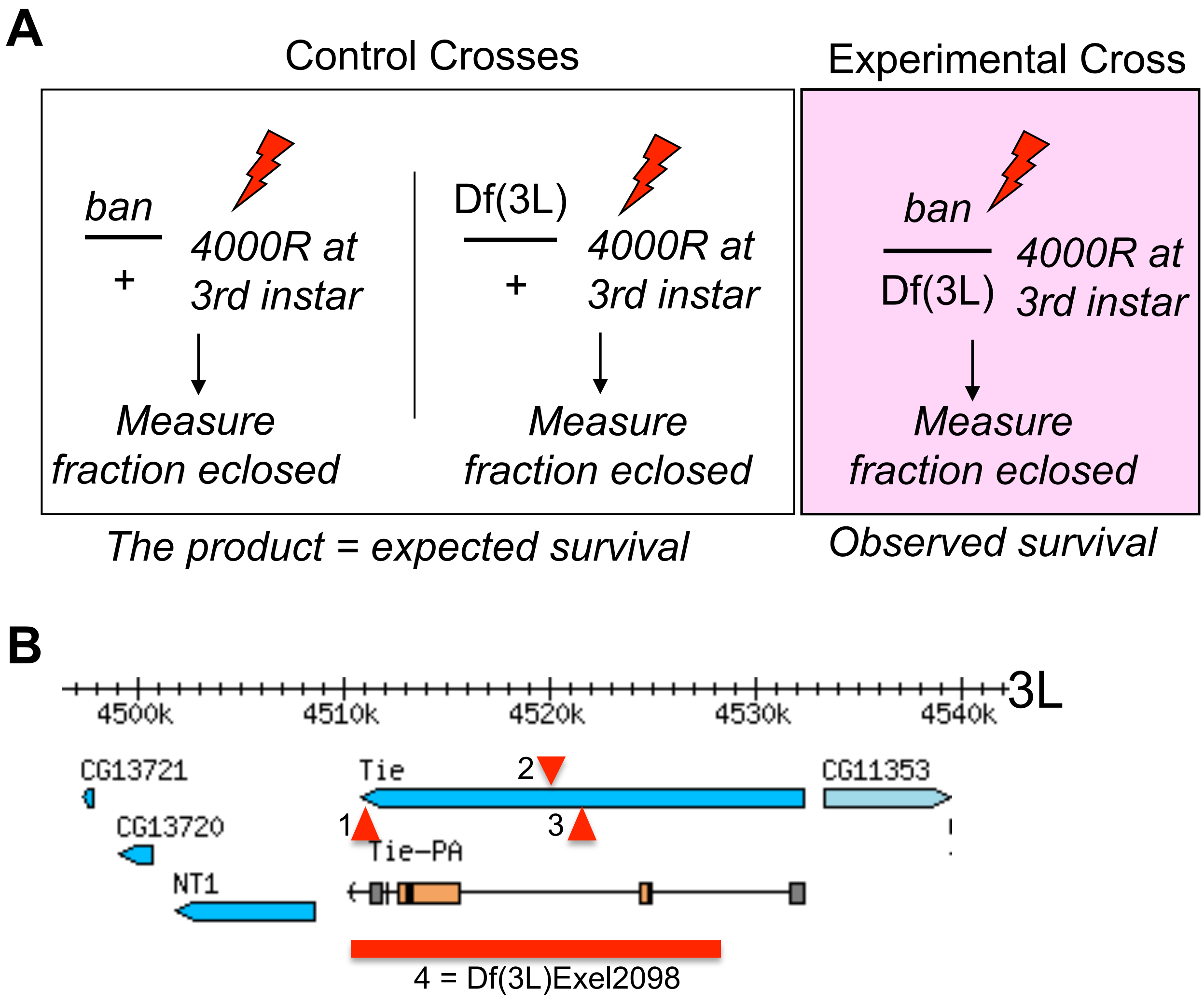

1 = PBAC{3HPy+}Tie^{c098}, inserted in predicted 3'UTR
2 = PBAC{RB}Tie^{e03394}, inserted in the second intron
3 = PBAC{RB}Tie^{e02680,} , inserted in the second intron
4 = Df(3L)Exel2098, predicted to remove the entire *tie* coding region and no other gene

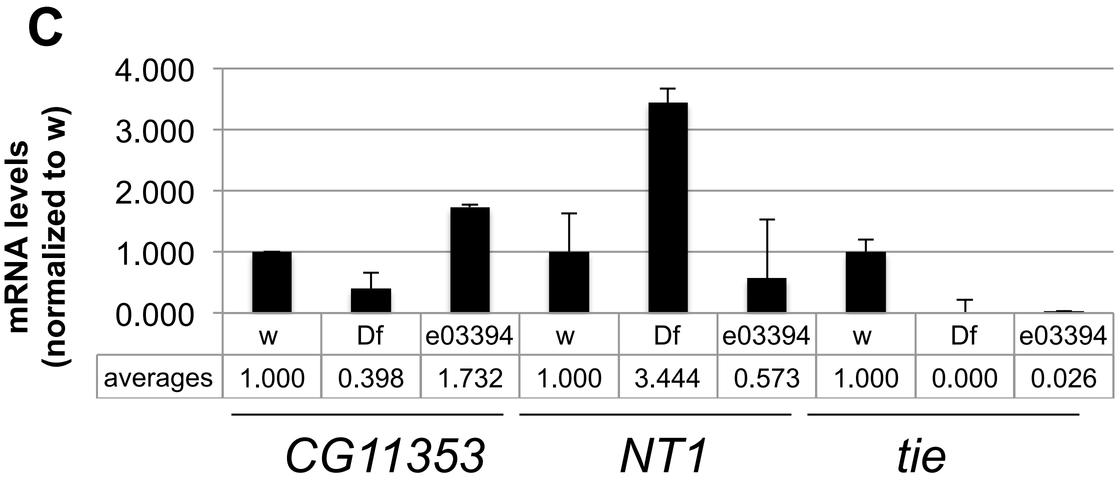

	w	Df	e03394	w	Df	e03394	w	Df	e03394
averages	1.000	0.398	1.732	1.000	3.444	0.573	1.000	0.000	0.026
