# Supplementary material for: Dying Cells Protect Survivors from Radiation-Induced Cell Death in Drosophila
Source: PLoS Genet. 2014 Mar 27;10(3):e1004220. doi: 10.1371/journal.pgen.1004220 (PMC3967929; doi:10.1371/journal.pgen.1004220)
Supplement: Table S1 — Changes in mRNA levels at 2 hours after IR. The data for pvf1, pvf2 and CG10359 are from published supplemental microarray data in Ref#30. The data for tie and pvf3 are from an RNAseq dataset (our unpublished data). (PDF) [file pgen.1004220.s011.pdf]

Bilak et al. Table S1

Table S1. Changes in the mRNA levels at 2 hours after IR

gene	y^1w^{1118}	p value	$p53^{5A-1-4}$	p value
pvf1	1.635	0.000013	-0.282	0.539042
pvf2	1.882	2.30E-13	0.137	0.377999
CG10359	1.452	0.000087	1.193	0. 042465
tie	1.280	0.692	ND	ND
pvf3	0.875	0.947	ND	ND
